# Oral Administration of East Asian Herbal Medicine for Inflammatory Skin Lesions in Plaque Psoriasis: A Systematic Review, Meta-Analysis, and Exploration of Core Herbal Materials

**DOI:** 10.3390/nu14122434

**Published:** 2022-06-12

**Authors:** Hee-Geun Jo, Hyehwa Kim, Donghun Lee

**Affiliations:** 1 BS Healthcare Co., Ltd., 11 Teheran-ro 33-gil, Gangnam-gu, Seoul 06141, Korea; 2 Department of Herbal Pharmacology, College of Korean Medicine, Gachon University, 1342 Seongnamdae-ro, Sujeong-gu, Seongnam 13120, Korea; hh1635@hanmail.net

**Keywords:** herbal medicine, psoriasis vulgaris, meta-analysis, cluster analysis, social network analysis, anti-inflammatory activity, synergistic effect, natural product, phytomedicine

## Abstract

Psoriasis is an inflammatory autoimmune skin disease with various clinical manifestations. The aim of this review was to systematically evaluate the efficacy and safety of oral administration of East Asian herbal medicine (EAHM) for inflammatory skin lesions in psoriasis and to explore core herbal materials for drug discovery. A comprehensive search was conducted in 10 electronic databases for randomized controlled trials from their inception until 29 July 2021. Statistical analysis was performed in R version 4.1.2 and R studio. When heterogeneity in studies was detected, the cause was identified through sensitivity analysis, meta-regression, and subgroup analysis. Methodological quality was independently assessed using the revised tool for risk of bias in randomized trials. A total of 56 trials with 4966 psoriasis patients met the selection criteria. Meta-analysis favored EAHM monotherapy on Psoriasis Area Severity Index (PASI) 70 (RR: 1.2845; 95% CI: 1.906 to 1.3858, *p* < 0.0001), PASI 60 (RR: 1.1923; 95% CI: 1.1134 to 1.2769, *p* < 0.0001), continuous PASI score (MD: −2.3386, 95% CI: −3.3068 to −1.3704, *p* < 0.0001), IL-17, IL-23, TNF-α, and Dermatology Life Quality Index. Patients treated with EAHM monotherapy had significantly reduced adverse events incidence rate. In addition, based on additional examination of the herb data included in this meta-analysis, 16 core materials were identified. They are utilized in close proximity to one another, and all have anti-inflammatory properties. The findings in this study support that oral EAHM monotherapy may be beneficial for inflammatory skin lesions in psoriasis. Meanwhile, the identified core materials are expected to be utilized as useful drug candidate hypotheses through follow-up studies on individual pharmacological activities and synergistic effects.

## 1. Introduction

Psoriasis is an inflammatory autoimmune skin disease with various clinical manifestations, and there are millions of these patients worldwide [1]. The prevalence of this disease is reported differently in each country, and the overall prevalence is known to be between 0.14% and 1.99% [2]. Most patients with psoriasis are exposed to very negative psychological effects due to skin findings in exposed areas, such as the face and limbs, as well as shortened life expectancy due to complications of the disease [3,4]. The seriousness of the problem is also highlighted by the research findings, which show that more than 20% of psoriasis patients are depressed, which can lead to suicidal conduct in severe situations [4,5]. In addition, recent studies have reported that psoriasis is associated with various chronic diseases that can negatively affect life expectancies, such as psoriatic arthritis, hypertension, type 2 diabetes, dyslipidemia, myocardial infarction, and stroke [1,6,7]. This means that psoriasis should be regarded as a systemic disease that can increase the social burden beyond a focal aesthetic problem for individual patients [8]. Therefore, it is a very important medical task at present to find a way to reduce the physical, social, and psychological problems caused by psoriasis through active medical management.

There are numerous clinical phenotypes of psoriasis, but plaque psoriasis, also known as psoriasis vulgaris, accounts for around 80% to 90% of cases [9]. Plaque can be expressed in a wide variety of thicknesses and sizes, and often appears as skin lesions accompanied by scales on the face, elbow, lumbosacral region, and scalp [1,9]. In mild cases where these plaques are less than 3–5% of the body surface, topical therapy or phototherapy can often be helpful [10]. However, for moderate-to-severe plaque psoriasis, oral systemic medications are required [1,10]. Oral agents that have been commonly used for severe plaque psoriasis include acitretin, apremilast, ciclosporin, methotrexate, etc. [10,11]. Recently, many biologics targeting a specific pathway of the immune system have been developed [11]. Even though many of these conventional medicines (CM) already exist, there are still problems that need improvement with respect to systemic therapy for psoriasis. For example, acitretin is contraindicated in women of childbearing age due to teratogenicity, and mild side effects such as dose-dependent hair loss and xerosis have been reported [9,12]. Meanwhile, methotrexate, which has been used for a long time, also has adverse effects such as hepatotoxicity and bone marrow suppression that can lead to cirrhosis [1,13]. Although biologics report improved effects compared to conventional oral drugs, there are still a not small proportion of patients who do not respond to medication at all. On the other hand, the cost of these drugs is also a significant factor that lowers adherence to treatment and lowers accessibility. Therefore, additional research on new drugs for the treatment of psoriasis with improved cost-effectiveness while having efficacy and safety not inferior to existing CMs is a subject of sufficient value.

East Asian herbal medicine (EAHM) refers to natural materials and theories used as medicines for the treatment of diseases in many countries in East Asia, including Korea, China, Taiwan, and Japan [14,15,16,17]. EAHM has a distinct prescribing principle that has been developed during many years of use [15,18]. In addition, it is distinctly different from natural materials in other regions of the world in that many of the same medicinal herbs appear in the pharmacopeia of East Asian countries. EAHM is not only being actively used in actual clinical practice, but also can be a useful resource for the discovery of new drugs based on accumulated experience and research [15,19,20]. For the treatment of psoriasis, a considerable amount of evidence on the efficacy and safety of EAHM has already been established through previous studies [21,22,23]. Looking at these, it is easy to confirm that EAHM offers evident therapeutic benefits in terms of the severity of psoriasis-related skin damage, and treatment response rate, and is a relatively safe intervention. Meanwhile, although the mechanism of psoriasis has not been fully elucidated, it is known that a wide variety of inflammation-related pathways are involved in pathogenesis. Given this, it is logical to expect EAHM, whose basic mechanism is a multi-component/multi-target action, to be helpful in modifying the immune system and systemic inflammatory states linked to psoriasis manifestation [18,24,25,26].

Despite the positive potential of EAHM for the treatment of psoriasis, there are problems to be solved first in the process of developing it into a useful drug. First of all, EAHM has the characteristic of being used in the form of a polyherbal formulation tailored to the individual patient’s findings, which is an important difference from herbal medicine in other regions of the world [14,27,28]. In this regard, EAHM’s pharmacological activity of individual herbs as well as the synergistic effect obtained from the combination of several herbs is a key therapeutic mechanism [18,24,28]. For this reason, it is not easy to select candidate materials with appropriate indications and mechanisms for the treatment of specific diseases among numerous EAHM. Narrowing the field of view to meta-analysis level evidence, several studies have dealt with the effects of EAHM monotherapy and EAHM and other intervention combination therapy simultaneously without distinguishing them. Moreover, in numerous studies verifying the effect of EAHM on psoriasis, discussions of various formulations and routes such as fumigation and ointments other than oral preparations are mixed. This suggests that it is difficult to see that the evidence for EAHM monotherapy with a specific route of administration has been established robustly. Therefore, at the present time, it is necessary to evaluate the efficacy and safety of EAHM for psoriasis based on a more rigorous study design for the route of administration and control group to be compared and to derive meaningful new drug candidate materials based on this data.

In accordance with the above recognition, we conducted a study according to the following objectives to provide clinicians with a clearer range of evidence, and at the same time, achieve the objective of exploring useful hypotheses for drug discovery: (1) efficacy and safety of EAHM monotherapy with the oral route of administration in inflammatory skin lesions of psoriasis are evaluated through the systematic review without limitation in scope. (2) Data mining on the herb data collected through this review is performed to derive a hypothesis related to the core EAHM material for psoriasis.

## 2. Methods

This study was conducted in accordance with the Preferred Reporting Items for Systematic Reviews and Meta-Analysis 2020 statement (Supplementary Table S1) [29]. The protocol of this systematic review was registered in PROSPERO (Registration Number: CRD42022296837, available from: https://www.crd.york.ac.uk/prospero/display_record.php?ID=CRD42022296837, accessed on 14 May 2022).

### 2.1. Search Strategy

Randomized controlled trials (RCT) that evaluated the efficacy and safety of EAHM monotherapy for plaque psoriasis were searched in the following 10 electronic databases from their inception until 29 July 2021: three English databases (PubMed, Cochrane Library, EMBASE), four Korean databases (Korean Studies Information Service System (KISS), Research Information Service System (RISS), Oriental Medicine Advanced Searching Integrated System (OASIS), Korea Citation Index (KCI)), two Chinese databases (Chinese National Knowledge Infrastructure Database (CNKI), Wanfang data), one Japanese database (CiNii). The following Boolean format was used for the search: (Psoriasis[Mesh]) AND ((Psoriases[Title/Abstract]) OR (Pustulosis of Palms[Title/Abstract] AND Soles[Title/Abstract]) OR (Pustulosis Palmaris et Plantaris[Title/Abstract]) OR (Palmoplantaris Pustulosis[Title/Abstract]) OR (Pustular Psoriasis of Palms[Title/Abstract] AND Soles[Title/Abstract])) AND (“Plants, Medicinal”[MeSH] OR “Drugs, Chinese Herbal”[MeSH] OR “Medicine, Chinese Traditional”[MeSH] OR “Medicine, Kampo”[MeSH] OR “Medicine, Korean Traditional”[MeSH] OR “Herbal Medicine”[MeSH] OR “Prescription Drugs”[MeSH] OR “traditional Korean medicine”[Title/abstract] OR “traditional Chinese medicine”[Title/abstract] OR “traditional oriental medicine”[Title/abstract] OR “Kampo medicine”[Title/abstract] OR herb*[Title/abstract] OR decoction*[Title/abstract] OR botanic*[Title/abstract]). In Korean, Chinese, and Japanese databases, these search terms were appropriately modified to perform a search. Detailed search strategies are explicated in Supplementary Table S2.

### 2.2. Study Selection

#### 2.2.1. Type of Studies

Only RCTs evaluating the efficacy and safety of oral administration of EAHM for plaque psoriasis were included. There were no restrictions on language and publication time. Some studies were excluded if they met the following criteria: (a) not RCT or quasi RCT; (b) not related plaque psoriasis or related disease; (c) primary intervention is not related EAHM; (d) not oral administration; (e) not clinical studies; (f) case reports or review; (g) not published in scientific peer-reviewed journals, including postgraduate theses or dissertations, and (h) when the experimental intervention is not EAHM monotherapy, such as combined therapy with conventional medicine.

#### 2.2.2. Type of Participants

Trials were considered eligible for inclusion if they were conducted in patients with psoriasis, with no restriction on age, gender, or race. Since the subject of this review is plaque psoriasis, clinical trials that include patients with other subtypes of psoriasis such as psoriatic arthritis, guttate psoriasis, palmoplantar pulposus, and erythrodermic psoriasis were excluded from the review.

#### 2.2.3. Type of Interventions

RCTs that compared EAHM as the active intervention in the treatment group versus placebo or CM in the control group were included. All forms of EAHM such as decoction, granule, capsule, compound preparation for the psoriasis treatment were included. There were no restrictions on the dose and duration of treatment for EAHM, but the mode of delivery was limited to oral intake. Studies in which East Asian medical interventions such as acupuncture, massage, or non-drug therapy were only combined in the treatment group were excluded. Studies in which the comparators included other EAHMs were excluded. Additionally, studies that were unable to verify the composition of specific herbal constituents that comprised the EAHM prescription utilized were omitted.

#### 2.2.4. Type of Outcome Measures

The response rate of patients whose psoriasis area severity index improved by greater than 60% (PASI 60) and 70% (PASI 70), respectively, was employed as the primary endpoint. Meanwhile, the absolute difference between groups in PASI score was also used as the primary outcome. Secondary outcomes include tumor necrosis factor *alpha* (TNF-α), Dermatology Life Quality Index (DLQI), Interlukin-17 (IL-17), Interlukin-23 (IL-23). In addition, to evaluate the safety of the intervention for psoriasis patients, the incidence of adverse events (AEs) was also included as a secondary outcome.

#### 2.2.5. Data Extraction

The titles and abstracts of potentially eligible studies were independently screened by 2 investigators (HGJ, HK) according to the above-mentioned search strategy. Afterward, a full-text review was performed based on the inclusion and exclusion criteria. Subsequently, information on the included studies was extracted independently by 2 reviewers (HGJ, HK). The following information was collected: title, author’s name, clinical trial conducted country, diagnostic criteria, trial design publication year, sample size, participant age, sex distribution, interventions in the treatment and comparators, treatment duration, outcome index, reported adverse event, and composition with the dosage of EAHM. Any discrepancy was discussed with the third author (DL).

#### 2.2.6. Methodological Quality Assessment

The methodological quality of each included study was evaluated independently by 2 investigators (HGJ, HK) according to the revised tool for risk of bias in randomized trials, Rob 2.0 [30]. It is comprised of five domains: bias arising from the randomization process, bias due to deviations from intended interventions, bias due to missing outcome data, bias in selection of the reported results. Methodological quality was assessed on three levels: “High risk of bias”, “Low risk of bias” and “Some concerns”. Disagreements between the two investigators were resolved with the help of the third author (DL).

#### 2.2.7. Statistical Analysis

##### Evidence Synthesis

Evidence synthesis of included studies with available data was performed by calculating the effect size and 95% CI using only the random effect model. Heterogeneity was considered statistically significant when the *p*-value based on the χ^2^ test was less than 0.10 or I^2^ was 50% or more. Two-sided *p* < 0.05 was considered statistically significant. Statistical synthesis of individual research results was performed in the software R version 4.1.2 and R studio program (Version 1.4.1106, Integrated Development for R. RStudio, PBC, Boston, MA, USA) using the default settings of the “meta” and “metafor” package [31]. The studies were grouped according to the type of intervention such as EAHM and comparator such as CM or placebo. Relative risk (RR) and 95% confidence interval (CI) were calculated for PASI 60 and PASI 70. Mean difference (MD) and 95% CIs were calculated for continuous PASI score and DLQI. For TNF-α, IL-17, and IL-23, standardized mean difference (SMD) and 95% CIs were calculated to integrate the results of several types of indicators for the same measurement target. Because the probability of an event that occurs was so much lower than other outcomes, and it was required to infer a causal relationship, AE was computed using odds ratio (OR). In this review, in order to effectively reveal the exact value of the effect size without relying only on the *p* < 0.05 significance threshold in the interpretation of the primary outcome synthesis result, a drapery plot was additionally illustrated along with the forest plot [32]. In the meta-analysis results, if heterogeneity was confirmed in an outcome that synthesized the results of more than 10 trials, the following additional analysis was performed to find out the cause. First, sensitivity analysis was performed according to the leave-one-out method to determine whether there was an effect by outliers in the included data. If no outliers are identified, after performing meta-regression analysis for the following three moderators specified in advance: (i) type of comparator, (ii) source of investigational medication, and (iii) type of EAHM formulation on the factors that had a substantial impact on the result, subgroup analyses were conducted. In order to distinguish publication bias, a contour-enhanced funnel plot was used for the outcome that included most studies [33]. For the asymmetry on the visually confirmed funnel plot, Egger’s test [34] and Begg’s test [35] were additionally performed to specifically confirm the existence of publication bias.

##### Hierarchical Agglomerative Clustering

The EAHM prescriptions used in each study reflect the medical goal of maximizing the synergy effect of the core herb combination. Therefore, hierarchical cluster analysis was used to understand the structure of the EAHM prescriptions used in individual studies. The analysis utilized in this study is agglomerative clustering, in which each observation is initially considered as a cluster of its own (leaf). Then, the most similar clusters are successfully merged until there is just one single big cluster (root).

The dissimilarity between individual herb constituents was considered as an individual distance, and the Euclidian distance was used as a measure of this. This corresponds to the shortest distance when it is assumed that the difference between each characteristic value is expressed on the coordinate plane.

(1) $$d\left(\chi_{i},\chi_{k}\right)=\sqrt{\overset{p}{\underset{j=1}{\sum }}{\left(\chi_{ij\;}-\chi_{kj\;}\right)}^{2}}$$

Cluster analysis in this study was performed on herbal constituents that showed a frequency of occurrence of at least 20% compared to the total included clinical trials.

##### Social Network Analysis

To explore the interdependence of fundamental herbal constituents utilized in the EAHM prescription and to uncover the core material of connection, a social network analysis was performed on the herb data of individual studies in this review. On the surface, the “complexity” discussed in social network analysis looks to be perplexing, yet it is a term that suggests that an order based on the interrelationships of the constituent pieces exists. EAHM’s prescription is an excellent illustration of the above-mentioned intricacy since it is guided by a combination of strict dosage principles and the tacit understanding of physicians who have worked with them for a long period. For this reason, the network analysis methodology has already been used in various ways in research analyzing EAHM [36,37].

Social network analysis in this review focused on two aspects. First, an undirected network was assumed, and the degree distribution was observed for the connectivity between the frequent herbal materials used in each EAHM prescription. In this case, since an undirected network is assumed, the average connection degree can be expressed as follows.

(2) $$A=\overset{n}{\underset{K=1}{\sum }}kP\left(k\right)=\frac{2E}{n}$$

(*n: number of nodes, E: number of links*)

Second, centrality was measured to identify herb materials with relatively large influence by comparing the influence of specific herbal medicines in the relationship between frequent herbs. Eigenvector centrality was used as the scale for the measurement that reflects the relationship between the individual herbs of EAHM that are prescribed at the same time. This scale can be expressed as:

(3) $$Ci=\frac{1}{\lambda }\underset{j\in N\left(i\right)}{\sum }A_{ij}C_{j}$$

λ is the eigenvalue of herb *i*, a constant measured by the algorithm, and N(*i*) is the set of neighboring herbs of herb *i*. *A_ij_* becomes “1” if herb *i* and *j* have a connection in the *n* × *n*-direction adjacency matrix *A*, and “0” if there is no connection. In the case of *C_j_*, it is the eigenvector centrality value of herb *j*, which is herb *i* and neighboring herbs.

#### 2.2.8. Quality of Evidence According to Outcome Measurements

The overall quality of evidence for each outcome was evaluated according to the Grading of Recommendations Assessment, Development, and Evaluation (GRADE) pro [38]. The GRADE assessment evaluates the overall quality of evidence in four levels: very low, low, moderate, and high. The level of evidence is lowered according to factors, such as the risk of bias, inconsistency, indirectness, imprecision, and publication bias, respectively.

## 3. Results

### 3.1. Study Identification

**Figure 1:**
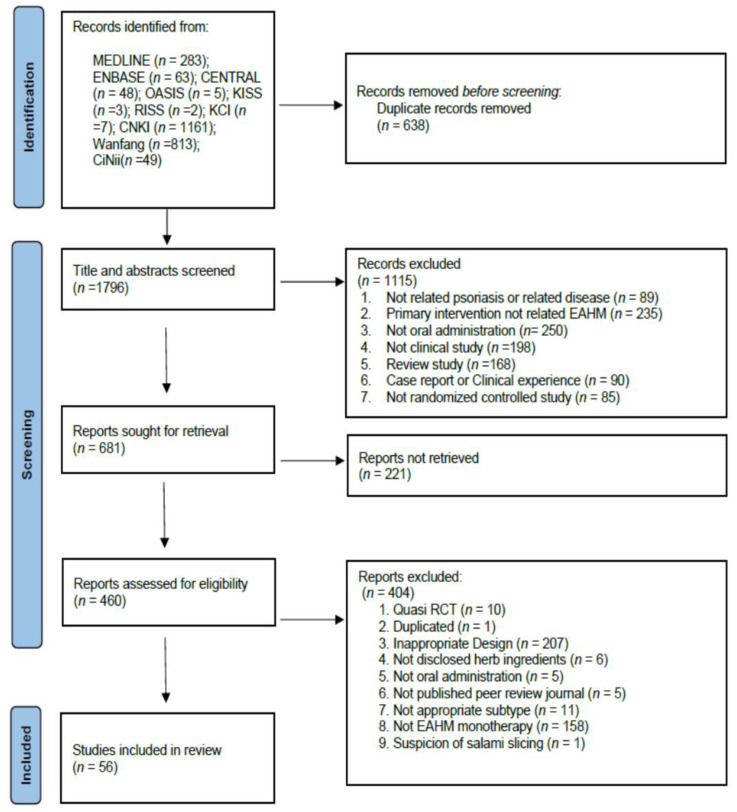
PRISMA 2020 flow diagram.

A total of 2434 studies were retrieved by electronic database search and manual search, among which 638 duplicate documents were removed. After screening the titles and abstracts, 1115 studies were excluded for at least one of the following reasons: (i) not related to psoriasis, (ii) primary intervention not related to EAHM, (iii) not oral administration, (iv) not clinical study (v) review article, (vi) case report or clinical experience, (vii) not a randomized controlled study. As a result of the evaluation of 460 articles for which full text was available among the remaining literature, 404 studies were excluded for the following reasons: (i) quasi-randomized controlled trials, (ii) duplicated documents, (iii) inappropriate study design, (iv) not disclosed herb ingredients, (v) not oral administration, (vi) not published peer-review scientific journal, (vii) not appropriate psoriasis subtype, (viii) not EAHM monotherapy, (ix) suspicion of salami slicing. Finally, 56 published studies were included in this review. Figure 1 shows the results of the database search.

### 3.2. Study Characteristics

The sample size of the included studies ranged from 40 to 260, and a total of 4966 participants were separated into the experimental group (n = 2605) and the control group (n = 2361). The psoriasis subtype in all included studies was psoriasis vulgaris or plaque psoriasis. One study was published in English, and all other studies were published in Chinese. The composition and formulation of the administered EAHM were reported in all studies included in this study. Only one study used a placebo preparation as a control group [39]; all other trials used CM as the control group. The following is a list of CMs that have been utilized as a control medication: methotrexate, vitamin A, glucocorticoids, and other topical medications including acitretin, compound amino-polypeptide agent, methotrexate, roxithromycin, penicillin, cephalosporin, vitamin A, glucocorticoids, and other topical agents. The duration of treatment in all eligible studies ranged from 2 weeks to 6 months. The characterization of the 56 included studies was summarized in detail in Table 1.

### 3.3. Risk of Bias

The methodological quality of 56 included studies was summarized in Table 2. The risk of bias in the studies was assessed by the Rob 2.0 tool [30]. The overall risk of bias in all studies was evaluated as “some concern”. This is related to the fact that domain 2, domain 4, and domain 5 were evaluated as “some concern” in all studies except for one study [56]. All studies evaluated as “some concern” in domain 2 and domain 4 did not employ a double-blind design, and it is unclear whether the outcome assessor and the interventionist were clearly separated. In addition to this, it was not possible to confirm the pre-registered protocol in all studies. Due to this common problem, the risk of bias could not be completely excluded in all studies.

### 3.4. Primary Outcomes

#### 3.4.1. PASI 70

**Figure 2:**
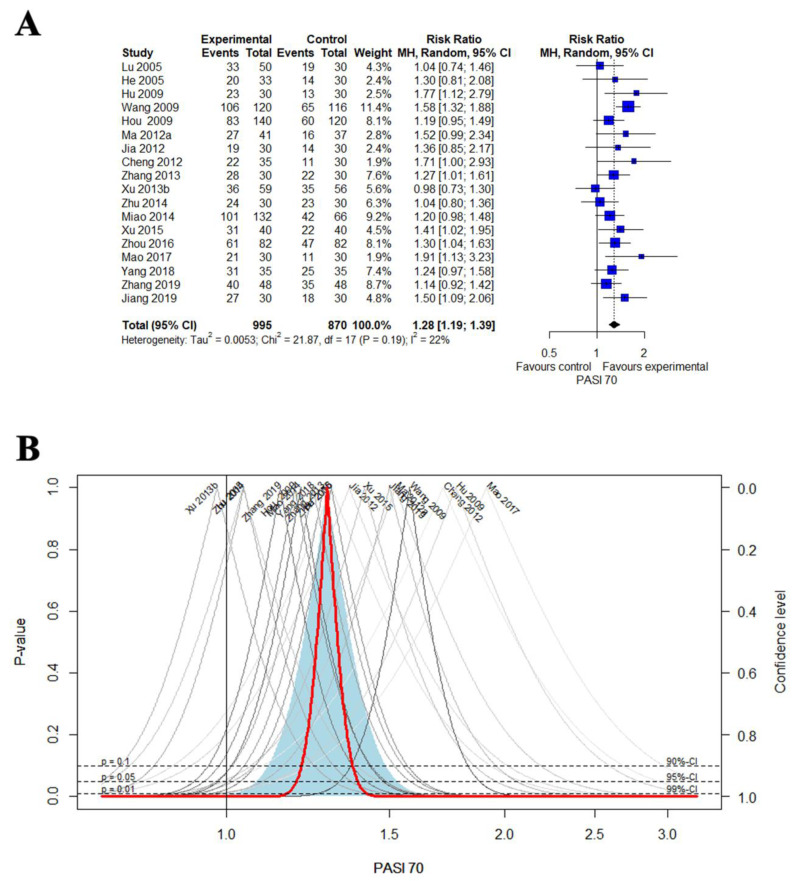
(**A**) Forest plot of the trials that compared EAHM with CM for PASI 70; (**B**) drapery plot of the trials that compared EAHM with CM for PASI 70.

A meta-analysis was performed on 18 studies that reported PASI 70 [43,45,51,52,55,62,63,64,66,69,70,75,76,83,86,89,91,92]. The combined results showed that EAHM had a statistically significantly better effect than the CM control group on the improvement of PASI 70 (18 trials, n = 1865; RR: 1.2845, 95% CI: 1.1906 to 1.3858, *p* < 0.0001; heterogeneity: χ^2^ = 21.87, df = 17, I^2^ = 22.3%, *p* = 0.1897; Figure 2A,B).

#### 3.4.2. PASI 60

**Figure 3:**
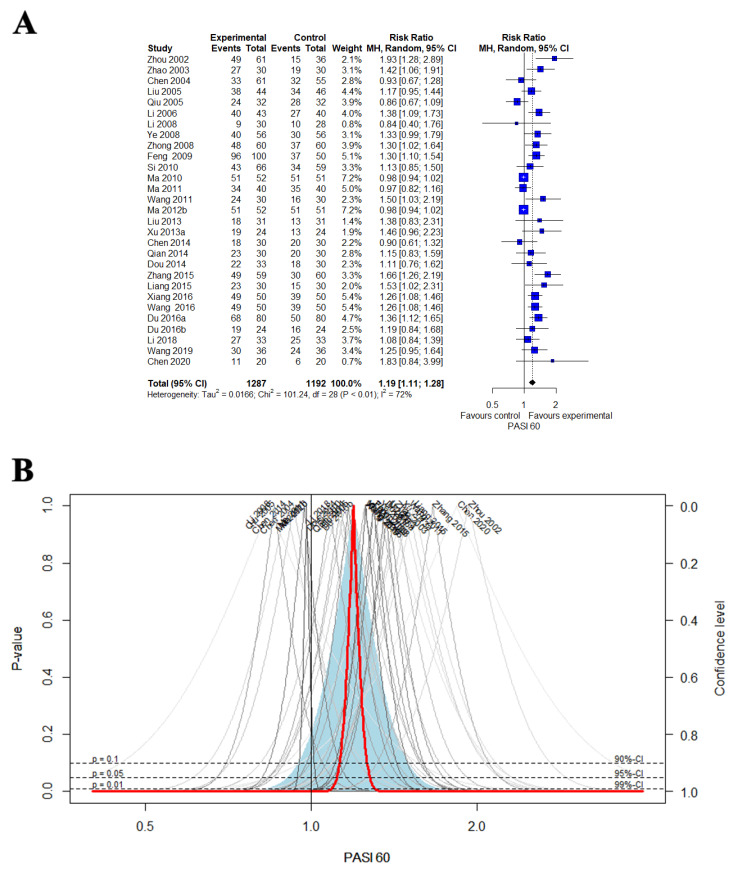
(**A**) Forest plot of the trials that compared EAHM with CM for PASI 60; (**B**) drapery plot of the trials that compared EAHM with CM for PASI 60.

A total of 29 studies compared EAHM with CM control regarding the PASI 60 [40,41,42,44,46,47,48,49,50,53,57,58,59,60,65,67,68,71,72,74,77,79,81,82,84,85,88,90,94]. The pooled effect of EAHM on the PASI 60 was significantly better than the CM control (29 trials, n = 2479; RR: 1.1923, 95% CI: 1.1134 to 1.2769, *p* < 0.0001; heterogeneity: χ^2^ = 101.24, df = 28, I^2^ = 72.3%, *p* < 0.0001; Figure 3A,B). Only one trial reported the effect of EAHM versus placebo control on PASI 60 [39]. Response rate in PASI 60 was significantly greater for EAHM than the placebo group (one trial, n = 56; RR: 3.7500, 95% CI: 1.4207 to 98983, *p* = 0.0076).

#### 3.4.3. Continuous PASI Score

**Figure 4:**
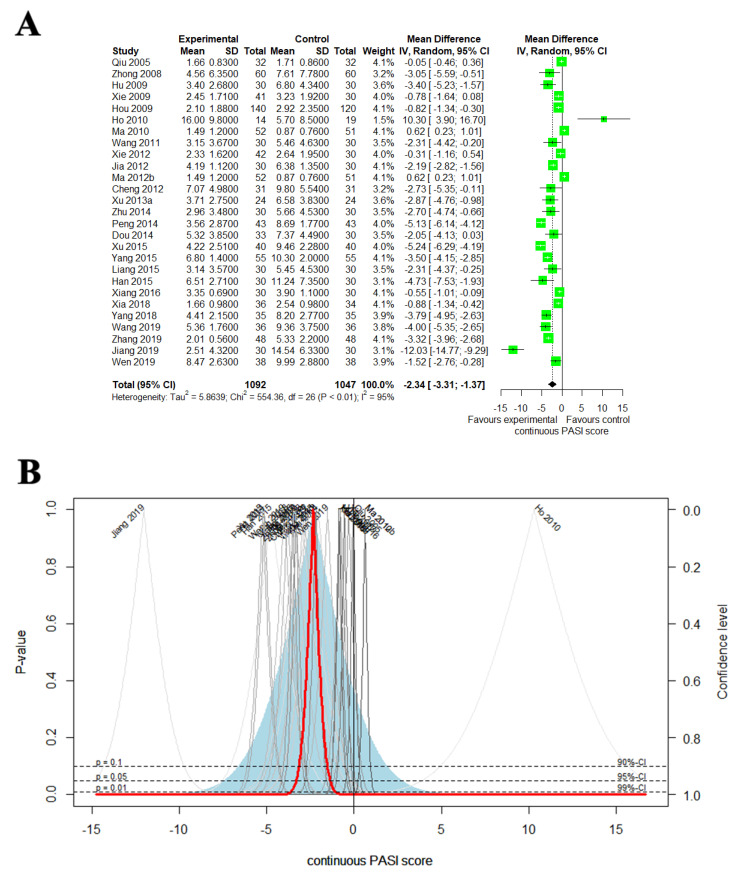
(**A**) Forest plot of the trials that compared EAHM with CM for continuous PASI score; (**B**) drapery plot of the trials that compared EAHM with CM for continuous PASI score.

In the 27 studies comparing the effect of EAHM with CM control, EAHM significantly improved continuous PASI score than CM control (27 trials, n = 2138; MD: −2.3386, 95% CI: −3.3068 to −1.3704, *p* < 0.0001; heterogeneity: χ^2^ = 554.36, df = 26, I^2^ = 95.3%, *p* < 0.0001; Figure 4A,B) [46,50,51,54,55,56,58,60,61,62,63,65,68,70,73,74,76,78,79,80,81,87,89,90,91,92,93].

### 3.5. Secondary Outcomes

#### 3.5.1. IL-17, IL-23, TNF-α and DLQI

**Figure 5:**
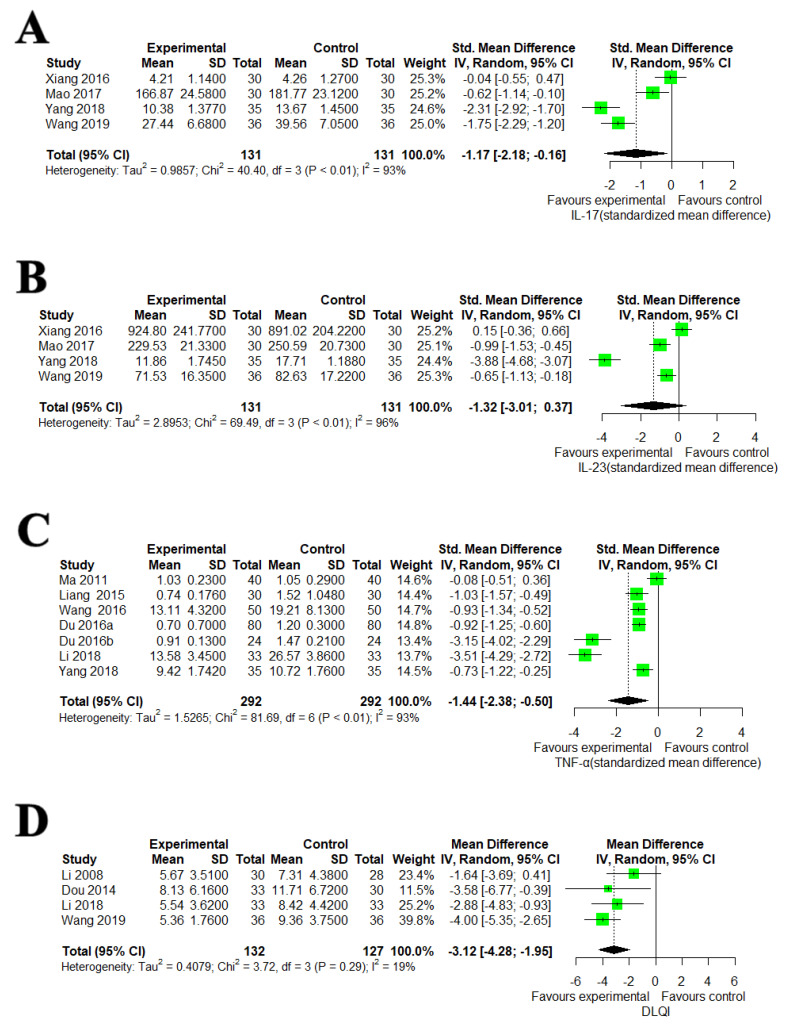
(**A**) Forest plot of the trials that compared EAHM with CM for IL-17, (**B**) forest plot of the trials that compared EAHM with CM for IL-23, (**C**) forest plot of the trials that compared EAHM with CM for TNF-α, (**D**) forest plot of the trials that compared EAHM with CM for DLQI.

Meta-analysis of four studies [81,86,89,90] showed that EAHMs were significant for reducing IL-17 compared to CM control (four trials, n = 262; SMD: −1.1683, 95% CI: −2.1789 to −0.1577, *p* = 0.0235; heterogeneity: χ^2^ = 40.40, df = 3, I^2^ = 92.6%, *p* < 0.0001; Figure 5A). IL-17 was also measured by the one trial that compared EAHM with placebo control [39]. A significant reduction in IL-17 level was observed by EAHM (one trial, n = 56; MD: −235.8200 pg/mL, 95% CI: −305.4477 to −166.1923, *p* < 0.0001). However, there is no significant difference between EAHM and CM control on IL-23 (four trials, n = 262; SMD: −1.3204, 95% CI: −3.0143 to 0.3734, *p* = 0.1265; heterogeneity: χ^2^ = 69.49, df = 3, I^2^ = 95.7%, *p* < 0.0001; Figure 5B). Seven studies compared the effect of EAHM to CM in reducing TNF-α [59,79,82,84,85,88,89]. Meta-analysis showed that EAHM significantly reduced TNF-α compared to CM control (seven trials, n = 584; SMD: −1.4396, 95% CI: −2.3803 to −0.4990, *p* = 0.0027; heterogeneity: χ^2^ = 81.69, df = 6, I^2^ = 92.7%, *p* < 0.0001; Figure 5C). DLQI was reported in four trials [48,74,88,90]. Compared with CM control, DLQI was significantly lower in the EAHM group (four trials, n = 259; MD: −3.1161, 95% CI: −4.2796 to −1.9526, *p* = 0.0001; heterogeneity: χ^2^ = 3.72, df = 3, I^2^ = 19.4 %, *p* = 0.2933; Figure 5D).

#### 3.5.2. AEs

**Figure 6:**
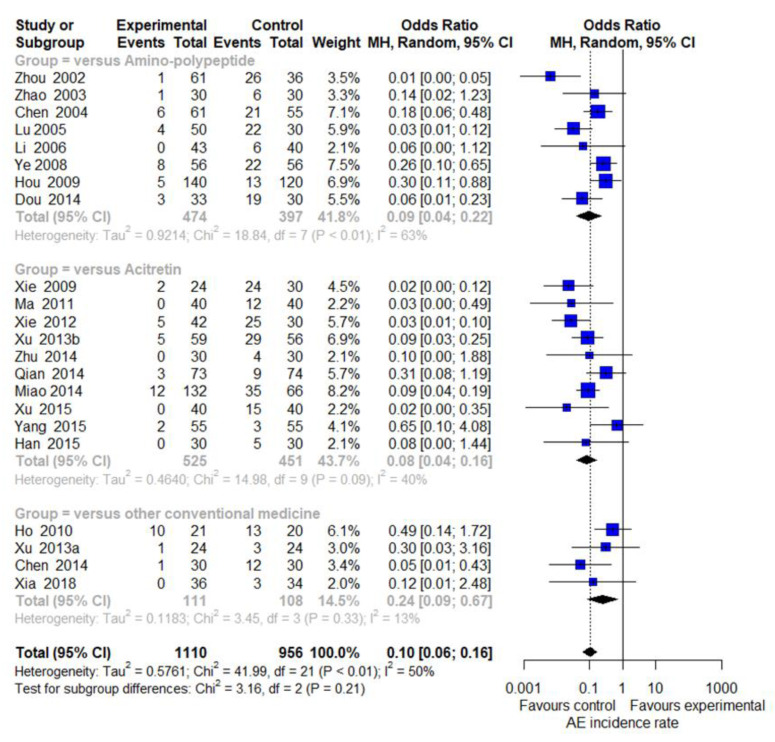
Forest plot for the incidence rates of reported adverse events.

Among the included studies, 33 trials (34/56, 60.71%) reported information related to AEs [40,41,42,44,46,47,49,50,51,54,55,56,58,59,61,62,63,65,67,68,69,70,71,72,74,75,76,77,78,80,84,87,92]. Four of these studies [46,50,58,77] did not report AEs in the control group, and two studies [62,65] reported the number of AEs in duplicate. On the other hand, there were five studies [51,63,67,84,92] that reported AEs in both groups. Therefore, 22 studies were able to synthesize the results by comparing the incidence rate. The aggregated results including 22 trials suggested that the incidences of AEs were significantly reduced by EAHM compared with CM control (22 trials, n = 2066; OR: 0.1017, 95% CI: 0.0630 to 0.1643, *p* < 0.0001; Figure 6). For the incidence rate of AEs, an additional comparison was performed through subgroup analysis according to the type of CM in the control group. Meta-analysis revealed that EAHM had lower incidence of AEs than amino-polypeptide agents (eight trials, n = 871; OR: 0.0939, 95% CI: 0.0399 to 0.2210, *p* < 0.0001; Figure 6). In comparison with acitretin, EAHM also showed a significant reduction in the incidence of AEs (10 trials, n = 976; OR: 0.0820, 95% CI: 0.0413 to 0.1628, *p* < 0.0001; Figure 6). Four studies comparing EAHM with other conventional medicines also showed a significant reduction in the incidence of AEs (four trials, n = 219; OR: 0.2428, 95% CI: 0.0879 to 0.6708, *p* < 0.0001; Figure 6). All the reported AEs were not severe and disappeared without long-term treatment. The details of adverse events reported in each study are recorded in Table 1.

### 3.6. Assessing Heterogeneity

#### 3.6.1. Sensitivity Analysis

**Figure 7:**
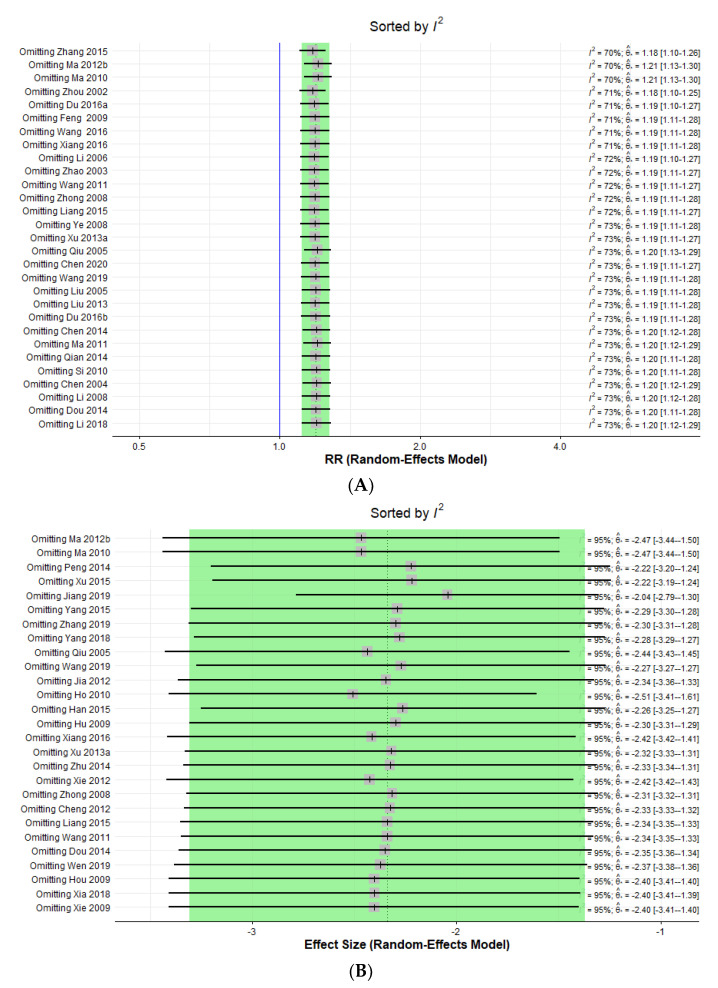
(**A**) Forest plot of the sensitivity analysis ordered by heterogeneity for PASI 60, (**B**) forest plot of the sensitivity analysis ordered by heterogeneity for continuous PASI score.

Considerable heterogeneity was found in the synthesis of trial data using PASI 60 and continuous PASI score outcomes, with I^2^ 72% and 95%, respectively. In the drapery plot, there were also studies that appeared to be outliers. Accordingly, sensitivity analysis was performed according to the leave-one-out approach to determine whether a specific study corresponding to these outliers was the cause of heterogeneity for the above two results. As a result of the sensitivity analysis, as shown in Figure 7, each omission for all individual studies did not have a noteworthy effect on heterogeneity change (Figure 7A,B).

#### 3.6.2. Meta-Regression and Subgroup Analysis

**Figure 8:**
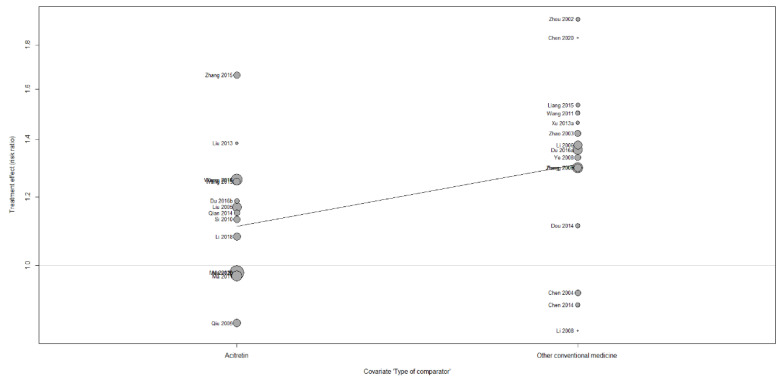
Bubble plot of PASI 60 for type of comparator.

Through sensitivity analysis, it was confirmed that outliers in individual studies did not affect heterogeneity. Hence, in order to identify other potential causes of heterogeneity, a meta-regression analysis was performed on moderators expected to influence the results. The moderators to be evaluated were “type of comparator”, “source of investigational medicine” and “sample size”, and they were applied to the meta-analysis findings of PASI 60 outcome and continuous PASI score, respectively, and analysis was performed. As a result of performing a meta-regression for PASI 60, the type of comparator that was confirmed as a variable had a statistically significant effect on the pooled results (*p* = 0.0104; Figure 8), but the source of investigational medicine (*p* = 0.6945; Supplemetary Figure S1) and sample size (*p* = 0.8941; Supplemetary Figure S2) did not have a statistically significant effect. Neither the type of comparator (*p* = 0.1902; Supplemetary Figure S3), the source of experimental medicine (*p* = 0.5499; Supplemetary Figure S4), nor the sample size (*p* = 0.4478; Supplemetary Figure S5) had a significant influence on the effect size of studies in a meta-regression of pooled results of continuous PASI score. Subgroup analysis indicated that the cause of heterogeneity may be related to the type of comparator (Table 3). Subgroup analysis was not performed for other predictors that were not significant in meta-regression. Meanwhile, for endpoints other than PASI 60 and continuous PASI score, additional sensitivity analysis, and subgroup analysis were not performed because the heterogeneity of the pooled results was low, or the number of included studies was very small.

### 3.7. Assessing Publication Bias

**Figure 9:**
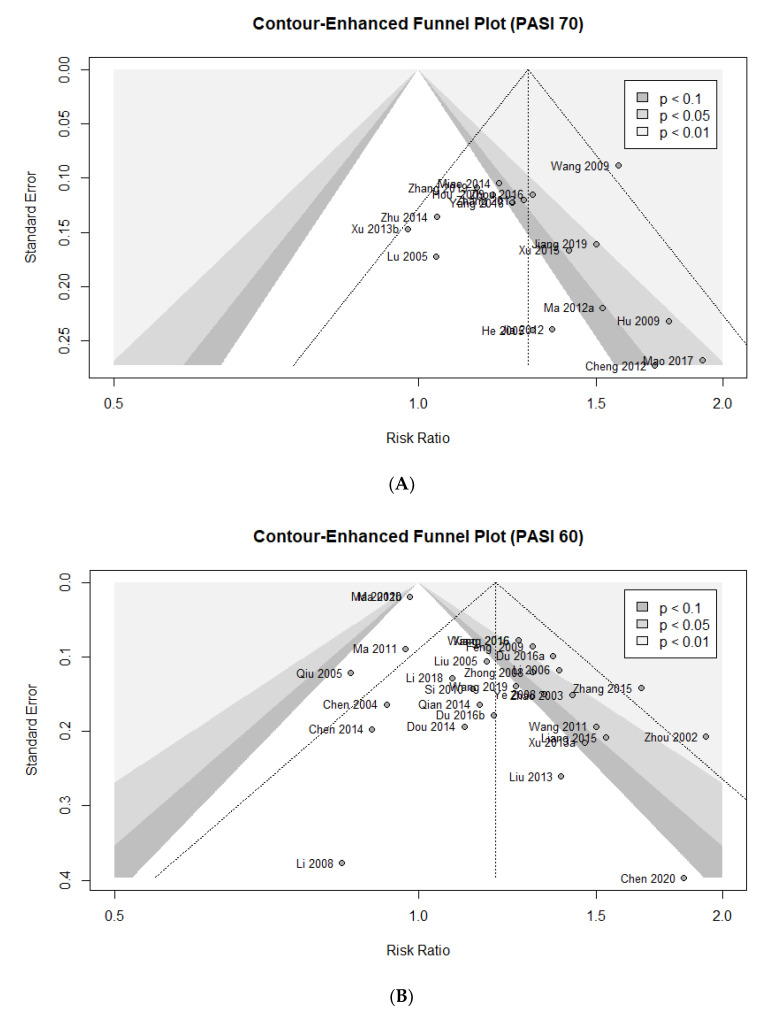

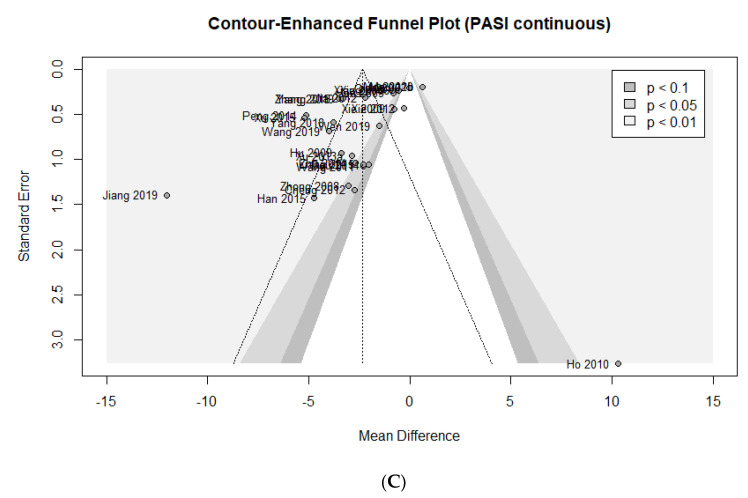
Contour-enhanced funnel plot of the trials for (**A**) PASI 70; (**B**) PASI 60; (**C**) continuous PASI score.

Contour-enhanced funnel plot, Egger’s test, and Begg’s test were used to assess the potential publication bias of the primary outcomes in this meta-analysis. Asymmetric shapes were observed in the contour-enhanced funnel plots for all outcomes, suggesting potential bias (Figure 9A–C). There was no evidence of significant publication bias in both Egger’s test and Begg’s test for PASI 70 (Egger’s test: *p* = 0.3501; Begg’s test: *p* = 0.1396). The publication bias was statistically significant in Egger’s test for PASI 60, but not in Begg’s test (Egger’s test: *p* < 0.0001; Begg’s test: *p* = 0.8511). Publication bias of continuous PASI score was also significant in Egger’s test, but no significant bias was confirmed in Begg’s test (Egger’s test: *p* = 0.0027; Begg’s test: *p* = 0.1038). Overall, there may be a risk of potential publication bias, but it is difficult to say that such findings have been confirmed very clearly. Although no unequivocal evidence showing publication bias was found in the above investigation, the risk of potential publication bias could not be fully eliminated.

### 3.8. Quality of Evidence According to Outcome Measures

In the comparison between EAHM and CM, the overall quality of evidence according to all outcome measures was very low to moderate. The results of the GRADE assessment are presented in Table 4.

### 3.9. Data Mining of EAHM Ingredients

#### 3.9.1. Detailed Information and Distribution of EAHM Ingredients

A total of 137 herbs were employed as component materials of the test EAHM in the 56 clinical trials covered in this review. Detailed information on individual EAHM components is summarized in Table 5. The following are 16 herbs that were prescribed with a high frequency in more than 20% of the studies included in this review: Rehmanniae Radix Recens; Salviae Miltiorrhizae Radix; Glycyrrhizae Radix et Rhizoma; Moutan Radicis Cortex; Lithospermi Radix; Smilacis Rhizoma; Radix Paeoniae Rubra; Dictamni Radicis Cortex; Imperatae Rhizoma; Hedyotidis Herba; Isatidis Radix; Lonicerae Flos; Sophorae Flos; Scutellariae Radix; Forsythiae Fructus; Spatholobi Caulis. The relative frequencies of these top 16 herbal materials ranged from 21.43% to a maximum of 69.64%. In terms of herb properties, all thirteen herbs, with the exception of three, were classed as cold and had the highest proportion, two herbs were neutral, and one herb had a warm property. Herbal flavors could be classed as bitter or sweet; however, bitter herbs accounted for a bigger part of the total, with nine herbs. Hence, the specific efficacy that clinicians consider when prescribing EAHM is expressed as summary information called the “action category”.

The action categories of the 16 high-frequency herbs mentioned above were all classified as “heat-clearing” except for 1. Table 6 shows the classification information for 16 herbs, including frequency distribution, property, taste, and action category.

#### 3.9.2. Hierarchical Agglomerative Clustering

**Figure 10:**
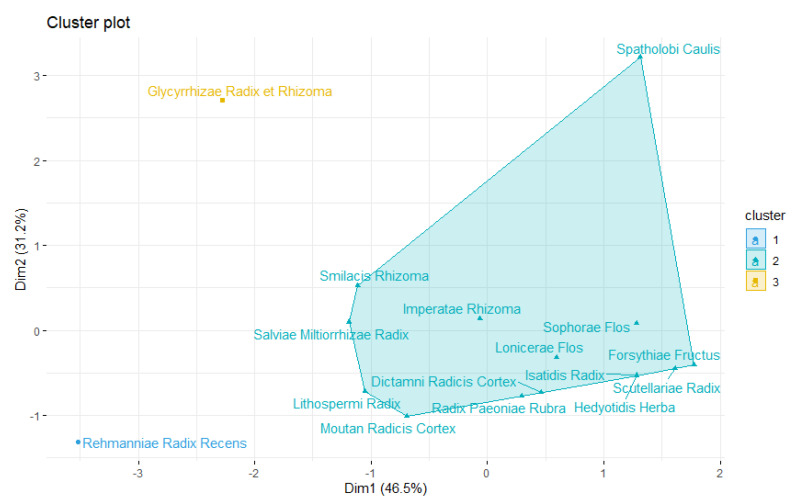
Scatter plot of 16 core EAHM material for treating psoriasis.

The characters of the top 16 high-frequency herbal materials were investigated using the hierarchical agglomerative cluster method. Through this analysis, pharmacological trends of core EAHMs used in the treatment of inflammatory skin lesions in psoriasis can be identified. The core herbs in this study may be separated into three modules as a result of classification based on the frequency of use and features of individual herbs. The results of classifying herbs are shown in Figure 10.

#### 3.9.3. Social Network Analysis

**Figure 11:**
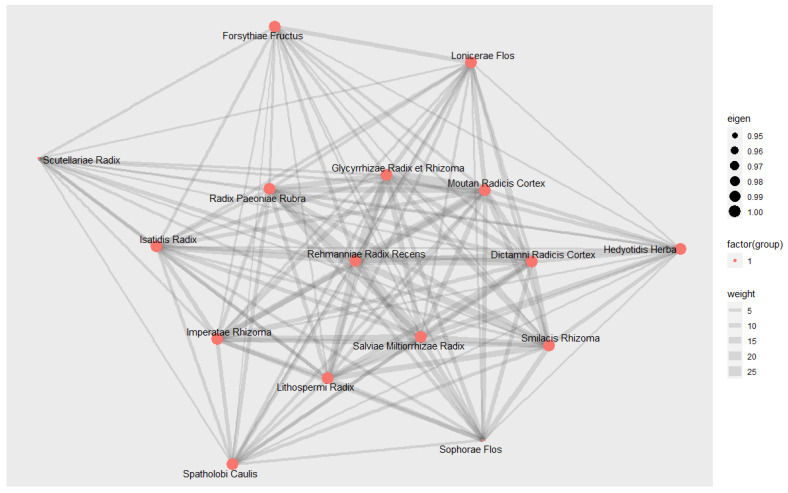
Core EAHM material network for treating psoriasis. The weight reflects the frequency at which two herbs are paired and utilized simultaneously in one EAHM prescription.

Social network analysis was used to confirm the mutual relationship between 16 herbs used frequently for inflammatory skin lesions of psoriasis and to identify core materials showing higher centrality in this interrelationship. As a result of graphically expressing the network between each herb, it was found that they are all closely connected, as shown in Figure 11. In the calculation of eigenvector centrality to measure the prestige centrality of individual herbs, Sophorae Flos and Scutellariae Radix were 0.0593, and all other 14 herbal materials were 0.0630. According to this, the centrality of 16 high-frequency herbs used in more than 20% of trials was generally at a similar level, and it could be interpreted that they were considered closely related to each other in their use in EAHM prescription for psoriasis.

## 4. Discussion

### 4.1. Summary of the Main Finding

Through the above analysis, our meta-analysis results suggest that oral EAHM is effective in improving symptoms of psoriasis. Overall, in the clinical trials included in this study, EAHM as monotherapy showed superior skin manifestation improvement in psoriasis compared to placebo and CM active controls in PASI 60, PASI 70, and continuous PASI indexes. At the same time, EAHM showed a superior or similar level of an effect to CM on the inflammatory findings of psoriasis in indicators such as IL-17, IL-23, and TNF-α, and also showed positive results on the quality of life in psoriasis. On top of that, patients treated that EAHM were more likely to experience less incidence rate of AEs. In this review, 16 high-frequency materials were derived through separate data mining of the collected herbal prescription information. Most of these herbs showed a clear tendency of property cold and action category “heat-clearing”, and it was found that all herbal materials were used with close correlation within the EAHM prescription.

### 4.2. Strength and Implications of Clinical Practice

The strength of this study is that we focused on the efficacy and safety of EAHM by the oral route of administration and as monotherapy alone. Since the efficacy that can be confirmed through clinical studies on combined therapy is an add-on effect, it should be viewed as essentially different from the efficacy of monotherapy of the intervention. On the other hand, even for materials with the same pharmacological effect, the fact that pharmacokinetics will vary depending on the administration route is no exception for natural products [95,96]. Recently, as the scope of research on pharmaceuticals based on natural sources continues to expand, the development of inhalation aerosol or injections is being actively carried out depending on the disease, as well as being used as external preparations such as ointment or fumigation [97,98,99]. Therefore, in the design of future EAHM studies, a clear definition of the administration route is bound to be a more important requirement. This study was aimed at deriving hypotheses related to candidate materials and indications for oral drugs beyond a simple meta-analysis, and there is no dispute that the route of administration and the conditions of monotherapy are important.

Evidence in this study derived according to the above scope suggests that oral administration of EAHM monotherapy is a useful option for inflammatory skin lesions management in psoriasis. The primary finding of this study is that the response rate and severity of PASI can be significantly improved. In addition, the improvement effect of various inflammation-related outcomes and DLQI is also a valuable finding in this study. These results are more meaningful in that they are consistent with several previous reports [21,23,100]. Therefore, administration of EAHM may be attempted as an indication for skin damage accompanied by inflammation in psoriasis patients. It seems reasonable to use EAHM for patients who show low compliance or do not respond to conventional CM treatment. Another important finding to consider is that when EAHM is used, the incidence of AEs is significantly reduced. Despite the need for systemic treatment through oral agents, it is worthwhile to apply EAHM monotherapy as an alternative to patients whose side effects of CM are too pronounced. Further analysis of the EAHM prescription data revealed that herb materials with specific properties were used frequently for psoriasis. Accordingly, the commonly prescribed core herbal material of this review and their close interrelationships information can help in the selection and combination of the appropriate herb when constructing customized EAHM formulations for individual patients.

### 4.3. Implications of Core Material Exploration

For the effective indications of EAHM for psoriasis revealed in the above discussion to be linked to the development of new drugs, further exploration of mechanisms and key materials is required. In this process, two important characteristics of EAHM must be considered first. One of them is related to the diagnostic method of East Asian medicine, which separately classifies the tendency to show systemic syndromes in addition to the patient’s biomedical symptoms and pathology [101]. Such a diagnostic method that can administer customized prescriptions for the same disease is called “pattern identification” or “syndrome differentiation”. The properties and action categories assigned to individual EAHM herbs represent therapeutic targets according to this diagnosis [27,102,103,104]. Specific EAHM indications have been primarily differentiated using the notions of “cold syndrome” and “hot syndrome,” and medications with “hot property/cold property” have been administered in response. For example, when a patient diagnosed with psoriasis complains of inflammatory skin symptoms along with physical findings such as fever, sweating, and thirst, it can be subdivided into hot syndrome of a psoriasis patient. EAHM materials that can effectively alleviate the accompanying systemic findings of this type of ‘hot syndrome’ are classified as cold properties. Conversely, EAHMs that can control cold syndrome are generally classified as hot property [105]. Recent studies exploring this topic at the molecular mechanism level have shown that EAHM, classified as a hot property, is implicated in pathways that include neurotransmitter reuptake, cold-induced thermogenesis, blood pressure regulation, and adrenergic receptor signaling. In the case of cold property, there are reports that the target gene is related to the steroid pathway. As a consequence, the hot/cold properties of EAHM were presumed to be the major factors in this study, implying distinct signals and mechanisms of action, which were incorporated in the analysis [106].

Most of the 16 high-frequency core herbs identified in this study were materials that exerted “clearing heat” action based on the “cold” property, and cluster analysis also confirmed that many herbs can be clustered with similar properties. This implies more information than simply that “clearing heat herb” is frequently used to manage inflammatory skin symptoms of psoriasis. According to previous studies, herbs exhibiting “clearing heat” action among EAHM are known to exhibit various anti-inflammatory and antiviral effects on patients with the so-called “heat” symptom pattern [107]. Hence, a more recent study revealed that “medicinal herbs of clearing heat” had multiple anti-inflammatory activities compared to herbs belonging to other action categories [108]. As summarized in Table 7, the pharmacological activity of the core herbs in this study is consistent with the knowledge in previous studies in that they correspond to anti-inflammatory and immune-modulating actions by various pathways. Therefore, the clinical efficacy of EAHM on psoriasis observed in this review appears to be strongly related to the complex anti-inflammatory mechanism exerted by herbs belonging to the “clearing heat” category. At the same time, in the future EAHM drug discovery related to psoriasis, it is expected that drugs corresponding to the above-discussed categories can be considered as preferred candidate materials.

On the other hand, another characteristic of EAHM that should be considered is the synergistic effect exerted through multi-compound action against the multi-target [28,125,126]. As can be seen from the data in this review, EAHM is usually administered in the form of a polyherbal formulation. This formulating chemical compound of EAHM not only produces a better synergistic effect, but also exerts an effect on the complex underlying mechanism of various diseases by reducing the side effects of individual drugs [18,127,128]. The main prescription principle of EAHM that makes this possible is expressed as “Gun-Shin-Jwa-Sa” (King-Retainer-Officer-Messenger in English words) [18,24]. In this approach, herbs responsible for the main effect are placed in a higher dose ratio at the positions of “Gun” and “Shin”, while herbs that lessen medication side effects or boost synergy are placed in relatively small doses at the positions of “Jwa” and “Sa”. Through this, an appropriately composed herbal combination can be expected to have amplified efficacy compared to that of a single herb. For example, the EAHM formula composed of only Sophorae Flos and Lonicerae Japonicae Flos, the high-frequency materials in this study, reprograms the immune microenvironment and exhibits anti-melanoma effects based on the mechanism that inhibits STAT3 signaling in B16F10 melanoma-bearing mice [129]. Meanwhile, Salvia Miltiorrhizae Radix, another core herb, and Notoginseng Radix et Rhizoma and 6:4 ratio were combined, and synergistic interaction was observed with respect to the protective effect of endothelial cells [130]. These previous studies suggest that rather than predicting the effect of EAHM only on the pharmacological activity of a single herb, considering the interaction between multiple materials together can bring better therapeutic outcomes. From this point of view, as a result of examining the relationship between the core herbs through social network analysis, close connectivity between all materials and an almost uniform level of betweenness centrality were observed. This finding supports the assumption that in the EAHM prescription of this study, the core herbs exerted an effect not only on the effect mechanism of individual herbs but also on the prescription composition principle according to the “Gun-Shin-Jwa-Sa” was considered by the application method. Therefore, tracking the synergy effect derived from the combination of key herbs and searching for the optimal herbal combination that can maximize this synergistic interaction can be a goal in follow-up studies for drug candidate proposals.

### 4.4. Limitations and Perspectives

To use the results and hypotheses derived from this study for clinical decision-making or follow-up research, it is necessary to understand the following limitations. First, as a result of performing a meta-analysis, a significant level of heterogeneity was observed. This suggests that it is difficult to accept that all EAHM prescriptions included in this study are useful for psoriasis. To investigate the cause of heterogeneity in detail, in this review, both outlier sensitivity analysis on individual trials and meta-regression on pre-specified moderators were performed. As a result, in the case of PASI 60, it was found that the type of CM adopted as an active control could be the cause, but in the case of a continuous PASI score showing a higher heterogeneity, a specific cause could not be identified. After excluding other causes, it could be presumed that the high heterogeneity was due to the very diverse composition and dosage of the EAHM prescription in each included trial. A similar problem is often seen in other meta-analyses of EAHM. This is due to EAHM’s prescription principle, which requires personalized prescription of herbal materials, and is highly likely to be repeated in future studies of the same design. Additional analysis of herbal material using data mining was performed as a way to overcome the essential limitations due to the characteristics of the intervention itself. If it is not a study that determines only natural products produced by pharmaceutical companies as the scope of analysis, it is thought that a separate analysis of EAHM prescriptions and herbal constituents by various methods in a systematic review related to EAHM in the future will be essential. Second, the commonly prescribed herb-related results derived from this review merely narrowed the scope of hypotheses about core materials through descriptive statistics and unsupervised learning techniques. Therefore, verification of whether the identified core herbs exert a better effect on psoriasis by themselves and whether actual synergy is created from the observed close correlation should be conducted through separate follow-up research. Based on the hypothesis presented in this study, it is thought that useful candidates can be further narrowed by comparing the effects of each EAHM through the network meta-analysis or predicting the mechanism using the network pharmacology technique together with the laboratory research. Third, as the primary outcome in this study, PASI 60 and PASI 70, which were adopted in the most inclusive studies, were selected as a relatively validated endpoint. However, considering that the evaluation instrument used as international standards in recent years is PASI 75 or PASI 90, it is difficult to completely rule out the bias in the results of this study as well. Therefore, in order to more objectively evaluate the efficacy of EAHM compared to placebo control as well as an active control, studies using widely used standard endpoints should be conducted. Fourth, most of the clinical trials included in this review lack pre-registered protocols, do not adopt double-blind methodologies and do not describe detailed randomization procedures. This shows that a number of studies cannot dissipate qualitative concerns, which will also affect the reliability of the results. Although the quantitative growth of EAHM-related evidence over the past decade has been remarkable, more clinical trials are still needed to ensure qualitative progress. Finally, a limitation is that all trials included in this study were conducted in China. In the process of collecting the literature for systematic review, there was no language restriction, and both databases in East Asia, as well as English databases, were searched, but only studies conducted in China met the inclusion criteria. However, as mentioned above, EAHM is widely used as a drug with a common material throughout East Asia, and the academic theory that is the principle of the application is also shared. Therefore, it is considered that the imbalance of trial execution regions is only due to differences in the medical research environment in each country. Therefore, it is expected that this difference can be overcome by continuously conducting studies such as this review on the usefulness of EAHM.

## 5. Conclusions

This systematic review supports that oral EAHM monotherapy can be a useful treatment for inflammatory skin lesions in psoriasis. Meta-analysis showed that EAHM had superior effects compared to the control group in PASI 70, PASI 60, continuous PASI score, IL-17, TNF-α, and DLQI of psoriasis patients. In addition, EAHM decreased the incidence rate of adverse events compared to the CM control group. In other words, it is thought that EAHM can positively contribute to skin symptoms, inflammatory status, quality of life, and drug adherence in psoriasis patients.

Further analysis of the EAHM prescription identified 16 high-frequency key materials: Rehmanniae Radix Recens; Salviae Miltiorrhizae Radix; Glycyrrhizae Radix et Rhizoma; Moutan Radicis Cortex; Lithospermi Radix; Smilacis Rhizoma; Radix Paeoniae Rubra; Dictamni Radicis Cortex; Imperatae Rhizoma; Hedyotidis Herba; Isatidis Radix; Lonicerae Flos; Sophorae Flos; Scutellariae Radix; Forsythiae Fructus; Spahalobi Caulis. They are generally thought to show multipath anti-inflammatory activity based on “heat clearing” action and show close connectivity. Therefore, in drug discovery related to this topic in the future, it is expected that the maximization of the anti-inflammatory synergy effect by the combination of EAHM materials belonging to the “heat clearing” category can be treated as a useful research hypothesis.

Despite the above results, concerns about the quality of the included studies and various biases were detected. To reach a firmer conclusion, additional clinical trials that include a multicenter design, a double-blind method, and an outcome with more validity in the design will need to be conducted in the future.

## Figures and Tables

**Table 1:** Characteristics of included studies.

First Author (Year) [Reference]	Type of Condition	Trial Design	Number of Participants (Male/Female); Age (Mean ± SD)		Interventions		Morbidity Period (Mean ± SD or Range)		Outcome Index	Course of Treatment	Adverse Event (Case/Symptom)
			Trial	Control	Trial	Control	Trial	Control			
Zhou (2002) [40]	Psoriasis vulgaris	Randomized; Single center; Parallel	61 (37/24) 36.5 y	36 (21/15) 38.7 y	Yuyin capsule (18 caps, t.i.d)	Compound amino-polypeptide tablets (10 caps, b.i.d)	4.7 y	4.3 y	1.PASI 60 response rate	8 w	Trial: 1 AE/ Control: 26 AEs/Thirst and xerostomia (8), xeroderma (7), desquamation (11)
Zhao (2003) [41]	Psoriasis vulgaris	Randomized; Single center; Parallel	30 (19/11) Range 19~63 y	30 (16/14) NR	Xiaoyin decoction (200 mL, t.i.d)	Compound Amino-polypeptide tablets (10 caps, b.i.d)	Range 0.5~45 y	Range 0.5~45 y	1.PASI 60 response rate	8 w	Trial: 1 AE/Loose stool Control: 6 AEs/ xerostomia, xeroderma
Chen (2004) [42]	Psoriasis vulgaris	Randomized; Single center; Parallel	61 (40/21) 37.51 ± 11.32 y	55 (35/20) 35.13 ± 10.91 y	Compound Qingdai pill (12 caps, t.i.d)	Compound Amino-polypeptide tablets (10 caps, q.d or b.i.d)	5.16 ± 5.02 y	4.87 ± 4.71 y	1.PASI 60 response rate	8 w	Trial: 6 AEs/Nausea and anorexia Control: 21 AEs/including xerostomia, xeroderma
Lu (2005) [43]	Psoriasis vulgaris	Randomized; Single center; Parallel	50 (32/18) 38.2 ± 16.4 y	30 (19/11) 39.3 ± 17.1 y	Yinxieling capsule (12~18 caps, t.i.d)	Compound Amino-polypeptide tablets (10 caps, b.i.d)	11.6 ± 8.4 y	11.3 ± 8.1 y	1. PASI 70 response rate	8 w	Trial: 4 AEs/Gastrointestinal reaction Control: 22 AEs/Xerostomia, xeroderma, scale, pruritus
Liu (2005) [44]	Psoriasis vulgaris	Randomized; Single center; Parallel	44 Both group (62/76) Range 17~64 y	46 Both group (62/76) Range 17~64 y	Jiedulaingxue decoction (b.i.d)	Etretin (30 mg, t.i.d)	NR	NR	1. PASI 60 response rate	4 w	Trial: No AE Control: pruritus and thirst (NR)
He (2005) [45]	Psoriasis vulgaris	Randomized; Single center; Parallel	33 (18/15) Range 16~65 y	30 (19/11) NR	1. Antidote decoction (b.i.d) 2. Tretinoin	1. Vitamin A [Retinol] (50,000 U, i.v., q.d) 2. Compound vitamin B tablets (6 caps, t.i.d) 3.Tretinoin	Range 2 m~30 y	Range 40 d~28 y	1. PASI 70 response rate	4 w	NR
Qiu (2005) [46]	Psoriasis vulgaris	Randomized; Single center; Parallel	32 (18/14) 30.42 ± 8.57 y	32 (17/15) 33.34 ± 8.21 y	Huoxuesanyuxiaoyin decoction (b.i.d)	Acitretin (20 mg, b.i.d)	6.53 ± 2.86 y	7.04 ± 3.12 y	1. PASI 60 response rate 2. PASI score	8 w	Trial: 3 AEs/Diarrhea (2), constipation and vomiting (1) Control: Total AEs NR/Xerostomia, xeroderma, scale, dizziness, headache/AST, ALT elevation (3)/BUN elevation (1)/hyperlipidemia (5)
Li (2006) [47]	Psoriasis vulgaris	Randomized; Single center; Parallel	43 (24/19) Range 13~55 y	40 (27/13) Range 15~67 y	1. Oral EAHM decoction (400 mL, b.i.d) 2. NB-UVB (0.3~0.5 J/cm^2^; 20%; NR; NR; q.o.d)	1. Compound Amino-polypeptide tablets (15 tabs, t.i.d) 2. NB-UVB (0.3~0.5 J/ cm^2^; 20%; NR; NR; q.o.d)	Range 1 m~42 y	Range 2 w~36 y	1. PASI 60 response rate	40 d	Trial: No AE Control: 5 AEs/Xerostomia, xeroderma, dizziness
Li (2008) [48]	Psoriasis vulgaris	Randomized; Single center; Parallel	30 (19/11) 42.16 ± 11.26 y	28 (18/10) 38.08 ± 9.64 y	Qinzhu Liangxue decoction (30 mL, b.i.d)	Compound amino-polypeptide tablets (15 tabs, t.i.d)	5.16 ± 1.34 y	6.28 ± 1.66 y	1. PASI 60 response rate 2. DLQI	4 w	NR
Ye (2008) [49]	Psoriasis vulgaris	Randomized; Single center; Parallel	56 (38/18) Range 8~65 y	56 (36/20) Range 9~68 y	Zhixuejieduxiaoyin decoction (b.i.d)	Compound amino -polypeptide tablets (15 tabs, t.i.d)	NR	NR	1. PASI 60 response rate	8 w	Trial: 8 AEs/ Dizziness, anorexia, abdominal distention, abdominal pain Control: 22 AEs/ xerostomia, hot flush, xeroderma, scale, pruritus
Zhong (2008) [50]	Psoriasis vulgaris	Randomized; Single center; Parallel	60 (39/21) 36.20 ± 10.74 y	60 (43/17) 36.18 ± 10.82 y	Xiaoyin granule (10.5 g, b.i.d)	Compound amino-polypeptide tablets (15 taps, t.i.d)	7.08 ± 4.46 y	7.24 ± 4.33 y	1. PASI 60 response rate 2. PASI score	8 w	Trial 12 AEs/Xerostomia, gastrointestinal discomfort, nausea, loose stool (12) Control: Total AEs NR Xerostomia (12), aggravated pruritus (22), dyssebacia (15), scale at hand and foot (9), dermatitis (8),conjunctival injection (2), hypermenorrhea (3)
Hu (2009) [51]	Psoriasis vulgaris	Randomized; Single center; Parallel	30 (16/14) 39.7 ± 11.7 y	30 (17/13) 37.0 ± 11.7 ye	Liangxue decoction (b.i.d)	Compound Amino-polypeptide tablets (15 tabs, t.i.d)	6.8 ± 3.4 y	6.7 ± 3.8 y	1.PASI 70 response rate 2.PASI score	8 w	Trial: No AE Control: No AE
Wang (2009) [52]	Psoriasis vulgaris	Randomized; Single center; Parallel	120 (65/55) Range 8~78 y	116 (62/54) Range 9~75 y	Baibi decoction (300 mL, b.i.d)	Compound Amino-polypeptide tablets (10 tabs, b.i.d)	Range 1 w~40 y	Range 1 w~35 y	1. PASI 70 response rate	90 d	NR
Feng (2009) [53]	Psoriasis vulgaris	Randomized; Single center; Parallel	100 (60/40) 43.28 ± 12.01 y	50 (30/20) 42.31 ± 11.08 y	Wushe decoction (q.d)	Compound econazole nitrate cream (b.i.d)	8.67 ± 6.5 y	8.23 ± 7.1 y	1. PASI 60 response rate	Trial: 30 d Control: 21 d	NR
Xie (2009) [54]	Psoriasis vulgaris	Randomized; Single center; Parallel	41 (21/20) Mean 42.5 y	30 (16/14) Mean 37.5 y	Kangyin1 decoction (b.i.d)	Acitretin (20 mg, b.i.d)	Range 1 m~28 y	Range 4 m~21 y	1. PASI score	8 w	Trial: 2 AEs/Gastrointestinal discomfort (2) Control: 24 AEs/Xeroderma (23), ALT elevation (2), hyperlipidemia (2), Headache with tinnitus (1), gastrointestinal discomfort (2)
Hou (2009) [55]	Psoriasis vulgaris	Randomized; Single center; Parallel	140 (72/68) 32.1 ± 6.6 y	120 (63/57) 38.4 ± 5.9 y	Huoxueliangxue decoction (b.i.d)	Compound Amino-polypeptide tablets (10 tabs, b.i.d)	Range 20 d~30 y	Range 15 d~32 y	1. PASI 70 response rate 2. PASI score	8 w	Trial: 5 AEs/diarrhea Control: 13 AEs/Xerostomia, (13), dizziness and drowsy (2)
Ho (2010) [56]	Plaque vulgaris	Randomized; Multi center; Parallel	21 (14/7) 48.52 y	20 (18/2) 43.45 y	Wen-tong- hua-yu formulation	1.Methotrexate (2.5~5 mg 1st week, increased to 10 mg q.w, not to exceed 30 mg q.w) 2.Folic acid (5 mg, q.d)	NR	NR	1. PASI score	24 w	Trial: 48% reported/Infection, gastrointestinal side effects, a few developed abnormalities in liver function Control: 65% reported/Nausea, vomiting, increased liver enzyme level
Si (2010) [57]	Psoriasis vulgaris	Randomized; Single center; Parallel	66 (28/38) 37.61 ± 14.43 y	59 (23/35) 34.25 ± 12.66 y	1. Jiawei Xiaoyaosan 2. Pulian ointment 3. NB-UVB (0.5 J/cm^2^; t.i.week)	1. Acitretin (20 mg, qd) 2. Pulian ointment 3. NB-UVB (0.5 J/cm^2^; t.i.week)	4.25 ± 5.06 y	3.40 ± 4.77 y	1. PASI 60 response rate	4 w	NR
Yan (2010) [39]	Psoriasis vulgaris	Randomized; Single center; Parallel	28 (Other information NR)	28 (Other information NR)	Quyin decoction (300 mL, b.i.d)	Placebo	NR	NR	1. PASI 60 response rate	12 w	NR
Ma (2010) [58]	Psoriasis vulgaris	Randomized; Single center; Parallel	52 (28/24) 39.04 ± 18.58 y	51 (26/25) 40.67 ± 13.64 y	Yinxiebing fang decoction (b.i.d)	Acitretin (30 mg, t.i.d)	4.82 ± 7.29 y	2.74 ± 3.32 y	1. PASI 60 response rate 2. PASI score	12 w	Trial: 5 AEs/Gastrointestinal discomfort (5) Control: Total AEs NR/Cheilitis (25), headache (6), tinnitus (2)/Gastrointestinal discomfort, liver function abnormality (4), xerostomia and scale (34), hyperlipidemia (8)
Ma (2011) [59]	Psoriasis vulgaris	Randomized; Single center; Parallel	40 (22/18) Mean 35.3 y	40 (23/17) Mean 37.8 y	Keyin Ⅰ prescription (300 mL, b.i.d)	Acitretin (30 mg, q.d; after 3rd week 60 mg, q.d)	7.8 y	8.3 y	1. PASI 60 response rate 2. TNF-α	90 d	Trial: No AE Control: 12 AEs/Cheilitis (3), pruritus and scale (7), Nausea with abdominal pain (2)
Wang (2011) [60]	Psoriasis vulgaris	Randomized; Single center; Parallel	30 (17/13) 35.24 ± 10.28 y	30 (16/14) 33.48 ± 10.02 y	Tufulingqingdai decoction (300 mL, b.i.d)	Compound Amino-polypeptide tablets (10 tabs, b.i.d)	6.5 y	5.4 y	1. PASI 60 response rate 2. PASI score	4 w	NR
Xie (2012) [61]	Psoriasis vulgaris	Randomized; Single center; Parallel	42 (22/20) Mean 41.5 y	30 (16/14) Mean 36.5 y	Liangxie Runfu decoction (b.i.d)	Acitretin (20 mg, b.i.d)	Range 1 m~25 y	Range 5 m~22 y	1. PASI score	12 w	Trial: 5 AEs/Gastrointestinal discomfort (5) Control: 25 AEs/Gastrointestinal discomfort (3), xerostomia and xeroderma (21), hyperlipidemia (1)
Jia (2012) [62]	Psoriasis vulgaris	Randomized; Single center; Parallel	30 (18/12) 35.67 ± 8.86 y	30 (16/14) 35.67 ± 8.86 y	Xiaobi decoction (300 mL, t.i.d)	Acitretin (20~30 mg, b.i.d or t.i.d)	8.13 ± 1.35 y	7.59 ± 1.46 y	1. PASI 70 response rate 2. PASI score	12 w	Trial: 9 AEs/Nausea (5), anorexia (2), loose stool (2) Control: 49 AEs/Nausea (6), anorexia (2), xeroderma (37), hyperlipidemia (4)
Cheng (2012) [63]	Psoriasis vulgaris	Randomized; Single center; Parallel	35 (13/22) Range 3~18 y	30 (10/20) Range 4~17 y	EAHM prescription for individual clinical trial (b.i.d)	1. Penicilin 2. Cephalosporin	NR	NR	1. PASI 70 response rate	4 w	Trial: No AE Control: No AE
Ma (2012a) [64]	Psoriasis vulgaris	Randomized; Single center; Parallel	41 (23/18) Mean 45.3 y	37 (21/16) Mean 44.8 y	Liangxue jiedu decoction (300 mL, b.i.d)	Compound Amino-polypeptide tablets (10 tabs, b.i.d)	Range 5 m~5 y	Range 5 m~5 y	1. PASI 70 response rate	8 w	NR
Ma (2012b) [65]	Psoriasis vulgaris	Randomized; Single center; Parallel	52 (28/24) 39.04 ± 18.58 y	51 (26/25) 40.67 ± 13.64 y	Yinxiaobing decoction	Acitretin (30 mg, t.i.d)	4.82 ± 7.29 y	2.74 ± 3.32 y	1. PASI 60 response rate 2. PASI score	12 w	Trial: 5 AEs/ Gastrointestinal discomfort (5) Control: 77 AEs/ Cheilitis (25), headache (6), tinnitus (2), abnormality of liver function (4), xeroderma and scale (34), hyperlipidemia (8)
Zhang (2013) [66]	Psoriasis vulgaris	Randomized; Single center; Parallel	30 (19/11) 40.83 ± 6.48 y	30 (18/12) 44.30 ± 5.80 y	Blood cooling decoction (300 mL, b.i.d)	Acitretin (20 mg, b.i.d)	6.24 ± 1.48 y	5.49 ± 1.24 y	1. PASI 70 response rate	8 w	NR
Liu (2013) [67]	Psoriasis vulgaris	Randomized; Single center; Parallel	31 (18/13) 40.55 ± 12.83 y	31 (16/15) 38.84 ± 10.57 y	Wanbi decoction (300 mL, b.i.d)	Acitretin (6 caps, b.i.d)	9.45 ± 5.07 y	7.80 ± 4.93 y	1. PASI 60 response rate 2. PASI score	Trial: 56 d Control: 60 d	Trial: No AE Control: No AE
Xu (2013a) [68]	Psoriasis vulgaris	Randomized; Single center; Parallel	24 (15/9) 44.78 ± 4.13 y	24 (16/8) 44.13 ± 4.46 y	1. Shufengyangtxue decoction (b.i.d) 2. Calcipotriol ointment (b.i.d)	1. Metotrexate (5 mg, b.i.d, continuous three days in a week) 2. Calcipotriol ointment (b.i.d)	101.53 ± 63.01 m	102.65 ± 63.01 m	1. PASI 60 response rate 2. PASI score	8 w	Trial: 1 AE/ Gastrointestinal discomfort (1) Control: 3 AEs/Gastrointestinal discomfort (3), loose stool (1)
Xu (2013b) [69]	Psoriasis vulgaris	Randomized; Single center; Parallel	59 (28/31) 41.26 ± 12.26 y	56 (26/30) 39.42 ± 10.87 y	Qingre Liangxue decoction (400 mL, b.i.d)	Acitretin (0.5 mg/kg, q.d)	6.24 ± 1.48 y	5.49 ± 1.24 y	1. PASI 70 response rate	6 w	Trial: 5 AEs/Gastrointestinal discomfort Control: 29 AEs/Gastrointestinal discomfort, cheilitis and scale
Zhu (2014) [70]	Psoriasis vulgaris	Randomized; Single center; Parallel	30 (14/16) 43.53 ± 2.15 y	30 (15/15) 43.75 ± 2.66 y	1. Dahuang Zhechong Capsule (4 caps, b.i.d) 2. Vitamin E cream (b.i.d)	1. Acitretin (30 mg, b.i.d−20 mg, 10 mg) 2. Vitamin E cream (b.i.d)	19.23 ± 2.33 y	18.17 ± 3.02 y	1. PASI 70 response rate 2. PASI score	12 w	Trial: No AE Control: 4 AEs/Xerostomia and xeroderma (3), elevation of liver enzyme (1)
Chen (2014) [71]	Psoriasis vulgaris	Randomized; Single center; Parallel	30 (16/14) 32.45 ± 24.89 y	30 (18/12) 31.73 ± 24.65 y	Liangxue No.1 formula (b.i.d)	1. Clobetasol propionate cream (t.i.d) 2. Loratadine (10 mg, q.d) 3. Acitretin (30 mg, q.d)	14.12 ± 4.76 y	14.58 ± 3.73 y	1. PASI 60 response rate	4 w	Trial: 1 AE/Nausea with vomiting Control: 12 AEs/Cheilitis, xerostomia, headache
Qian (2014) [72]	Psoriasis vulgaris	Randomized; Single center; Parallel	Both group 74 (43/31) 23.1 ± 2.6 y Trial: 38	Both group 74 (43/31) 23.1 ± 2.6 y Control: 36	1. Liangxue Runfu decoction (b.i.d) 2.15% urea cream (b.i.d)	1. Acitretin (20 mg, b.i.d) 2.15% urea cream (b.i.d)	5.2 ± 1.8 y (Both group)	5.2 ± 1.8 y (Both group)	1. PASI 60 response rate	8 w	Trial: 3 AEs/Gastrointestinal discomfort (3), xeroderma (2) Control: 9 AEs/Xerostomia (2), xeroderma (4), hyperlipidemia (2), gastrointestinal discomfort (1)
Peng (2014) [73]	Psoriasis vulgaris	Randomized; Single center; Parallel	Both group 86 (45/41) 51.36 ± 4.22 y Trial: 43	Both group 86 (45/41) 51.36 ± 4.22 y Control: 43	Liangxue Jiedu decoction (300 mL, b.i.d)	Compound amino-polypeptide tablets (15 tabs, t.i.d)	9 ± 3.2 y (Both group)	9 ± 3.2 y (Both group)	1. PASI score	12 w	NR
Dou (2014) [74]	Psoriasis vulgaris	Randomized; Single center; Parallel	33 (21/12) 38.6 ± 11.9 y	30 (19/11) 36.2 ± 12.5 y	1. Wutengxiaoyin Decoction 2.10% urea cream (b.i.d)	1. Compound amino-polypeptide Tablets (15 tabs, t.i.d) 2.10% urea cream (b.i.d)	13.4 ± 12.5 y	14.3 ± 8.7 y	1. PASI 60 response rate 2. PASI score 3. DLQI	8 w	Trial: 3 AEs/Gastrointestinal discomfort (2), diarrhea (3) Control: 19 AEs/ Xeroderma, xerostomia, scale (19), pruritus (4)
Miao (2014) [75]	Psoriasis vulgaris	Randomized; Single center; Parallel	Both group 198 (118/80) 39.6 ± 8 y Trial: 132	Both group 198 (118/80) 39.6 ± 8 y Control: 66	Quyin decoction (b.i.d)	Acitretin (10 mg, t.i.d)	Mean 3.9 y (Both group)	Mean 3.9 y (Both group)	1. PASI 70 response rate	12 w	Trial: 12 AEs/Headache with dizziness (8), nausea and vomiting (6), liver function abnormality (3) Control: 35 AEs/ Xerostomia (25), xerophtalmia (18), xeroderma (14), pruritus (6), headache and dizziness (6), ALT elevation (8)
Xu (2015) [76]	Psoriasis vulgaris	Randomized; Single center; Parallel	40 (23/17) 58.6 ± 8.8 y	40 (19/21) 59.8 ± 9.3 y	Liangxue Jiedu Decoction (300 mL, t.i.d)	Acitretin (25 mg, b.i.d)	12.5 ± 2.6 y	13.7 ± 2.1 y	1. PASI 70 response rate 2. PASI score	8 w	Trial: No AE Control: 15 AEs/Xerostomia, gastrointestinal discomfort (6), pruritus and scale (9)
Zhang (2015) [77]	Psoriasis vulgaris	Randomized; Single center; Parallel	60 (38/25) 31.29 ± 0.04 y	65 (35/30) Mean 29.22 y	Ziyinqingrexiaofeng san (NR)	1. Acitretin (20 mg, b.i.d) 2. Compound Flumetasone ointment (b.i.d)	Range 3 m~10 y	Range 1~12 y	1. PASI 60 response rate	8 w	Trial: 8 AEs/Burningsensation (5), erythema (2), aggravated pruritus (1) Control: NR
Yang (2015) [78]	Psoriasis vulgaris	Randomized; Single center; Parallel	55 (32/23) 37.6 ± 3.3 y	55 (30/25) 37.9 ± 3.5 y	Qingre liangxue decoction (b.i.d)	Acitretin (0.5 mg/kg, q.d)	8.8 ± 0.7 y	8.6 ± 0.5 y	1. PASI score	4 w	Trial: 2 AEs/Gastrointestinal discomfort (2) Control: 3 AEs/ Xerostomia (2), scale (1)
Liang (2015) [79]	Psoriasis vulgaris	Randomized; Single center; Parallel	30 (16/14) 34.28 ± 10.26 y	30 (18/12) 33.46 ± 10.12 y	1. Tufuling Qingdai decoction (300 mL, b.i.d) 2.Vaseline (b.i.d)	1. Compound amino-polypeptide tablets (10 tabs, b.i.d) 2. Vaseline (b.i.d)	Mean 6.8 y	Mean 5.6 y	1. PASI 60 response rate 2. PASI score 3. TNF-α	4 w	NR
Han (2015) [80]	Psoriatic pustules	Randomized; Single center; Parallel	30 (17/13) 37.71 ± 12.8 y	30 (16/14) 36.48 ± 12.34 y	Huayin Jiedu decoction (b.i.d)	Acitretin (20 mg, b.i.d)	8.64 ± 5.43 y	8.51 ± 7.89 y	1. PASI score	8 w	Trial: No AE Control: 5 AEs/Elevated triacylglycerols
Xiang (2016) [81]	Psoriasis vulgaris	Randomized; Single center; Parallel	30 (16/14) 37.5 ± 7.5 y	30 (17/13) 37.8 ± 7.2 y	Qinzhu Liangxue decoction (400 mL, b.i.d)	Acitretin (20~30 mg, b.i.d or t.i.d)	10.23 ± 7.2 y	11.23 ± 8.2 y	1. PASI score 2.IL-17 3.IL-23	4 w	NR
Wang (2016) [82]	Psoriasis vulgaris	Randomized; Single center; Parallel	50 (28/22) 27.2 ± 5.2 y	50 (27/23) 27.3 ± 6.2 y	Liangxue Runfu decoction (t.i.d)	Acitretin (10 mg, b.i.d)	NR	NR	1. PASI 60 response rate 2. TNF-α	NR	NR
Zhou (2016) [83]	Psoriasis vulgaris	Randomized; Single center; Parallel	82 (46/36) 35.7 ± 9.4 y	82 (48/34) 36.2 ± 9.7 y	Shufeng jiedu capsules (12 caps, t.i.d)	Compound amino-polypeptide tablets (6 tabs, b.i.d)	4.2 ± 2.1 y	4.1 ± 2.2 y	1. PASI 70 response rate	12 w	NR
Du (2016a) [84]	Psoriasis vulgaris	Randomized; Single center; Parallel	80 (56/24) 47.3 ± 10.3 y	80 (56/21) 48.7 ± 13.3 y	Shengdi Baimao decoction (300 mL, b.i.d)	0.025% Tretinoin ointment (b.i.d)	2.3 ± 1.8 y	2.5 ± 1.7 y	1. PASI 60 response rate 2. TNF-α	20 d	Trial: No AE Control: No AE
Du (2016b) [85]	Psoriasis vulgaris	Randomized; Single center; Parallel	24 (14/10) 41.75 ± 9.03 y	24 (15/9) 42.11 ± 10.95 y	Heat-clearing and detoxicating oral liquid (60 mL, t.i.d)	Acitretin (30 mg, t.i.d)	6.7 ± 4.4 y	7.1 ± 5.2 y	1. PASI 60 response rate 2. TNF-α	12 w	NR
Mao (2017) [86]	Psoriasis vulgaris	Randomized; Single center; Parallel	30 (21/9) 48.96 ± 6.88 y	30 (22/8) 48.02 ± 7.18 y	1. Xiaoyinfang (200 mL, b.i.d) 2. NB-UVB	1. Calcipotriol ointment (b.i.d) 2. NB-UVB	3.77 ± 1.27 y	3.62 ± 1.07 y	1. PASI 70 response rate 2. IL-17 3. IL-23	8 w	NR
Xia (2018) [87]	Psoriasis vulgaris	Randomized; Single center; Parallel	36 (21/15) 37.25 ± 13.44 y	34 (23/11) 34.85 ± 12.01 y	Kanli fang (400 mL, b.i.d)	0.005% Calcipotriol ointment (q.d)	7.57 ± 7.25 y	7.57 ± 7.25 y	1. PASI score	6 w	Trial: No AE Control: 3 AEs/Burning sense (3)
Li (2018) [88]	Psoriasis vulgaris	Randomized; Single center; Parallel	33 (19/14) 35.52 ± 3.72 y	33 (18/15) 35.48 ± 3.62 y	Jianpi Jiedu decoction (400 mL, b.i.d)	Acitretin (40 mg, b.i.d)	6.42 ± 3.65 y	6.35 ± 3.45 y	1. PASI 60 response rate 2. DLQI 3. TNF-α	NR	NR
Yang (2018) [89]	Psoriasis vulgaris	Randomized; Single center; Parallel	35 (18/17) 33.89 ± 2.68 y	35 (17/18) 34.26 ± 2.91 y	Jinji Xiaoyin granule (27 g, t.i.d)	Acitretin (40 mg, b.i.d)	3.62 ± 3.21 y	3.26 ± 3.42 y	1. PASI 70 response rate 2. PASI score 3. IL-17 4. IL-23 5.TNF-α	8 w	NR
Wang (2019) [90]	Psoriasis vulgaris	Randomized; Single center; Parallel	36 (20/16) 41.4 ± 3.5 y	36 (18/18) 39.2 ± 2.4 y	Qingying decoction (300 mL, b.i.d)	Acitretin (20 mg, b.i.d)	15.1 ± 3.5 y	14.2 ± 27 y	1. PASI 60 response rate 2. PASI score 3. DLQI 4. IL-17 5. IL-23	12 w	NR
Zhang (2019) [91]	Psoriasis vulgaris	Randomized; Single center; Parallel	48 (28/20) 36.84 ± 6.20 y	48 (28/20) 36.60 ± 6.20 y	Xijiao Dihuang decoction (300 mL, b.i.d)	Acitretin (20 mg, b.i.d)	56.53 ± 12.30 m	57.20 ± 12.50 m	1. PASI 70 response rate 2. PASI score	12 w	NR
Jiang (2019) [92]	Psoriasis vulgaris	Randomized; Single center; Parallel	30 (18/12) 20.15 ± 2.55 y	30 (14/16) 15.34 ± 4.71 y	1. Xijiao Dihuang Jiedu decoction (q.d) 2. Urea ointment for external use (b.i.d.)	1. Roxithromycin (Adlut: 300 mg, b.i.d; Adolescent: 2.5~5 mg*kg, b.i.d) 2. Urea ointment for external use (b.i.d.)	NR	NR	1. PASI 70 response rate 2. PASI score	2 w	Trial: No AE Control: No AE
Wen (2019) [93]	Psoriasis vulgaris	Randomized; Single center; Parallel	38 (26/12) 33.42 ± 8.37 y	38 (23/15) 31.44 ± 7.42 y	Xiaobi decoction (b.i.d)	Acitretin (40 mg, b.i.d)	46.87 ± 15.10 m	43.07 ± 15.98 m	1. PASI score	4 w	NR
Chen (2020) [94]	Psoriasis vulgaris	Randomized; Single center; Parallel	20 (11/9) 33.65 ± 5.41 y	20 (12/8) 33.58 ± 5.26 y	1. Quyin decoction (200 mL, b.i.d) 2.10% Urea ointment	1. Compound amino poly-peptide tables (10 tabs, b.i.d) 2. 10% Urea ointment	10.25 ± 3.35 y	10.19 ± 3.59 y	1. PASI 60 response rate	NR	NR

AE: Adverse event; b.i.d: bis in die; d: days; DLQI: Dermatology Life Quality Index; i.v: intravenous; IL-17: Interlukin-17; IL-23: Interlukin-23; m: months; mg: milligram; mL: milliliter; NR: Not reported; p.o: per os; PASI; psoriasis area severity index; q.d: quaque die; q.o.d: quaque altera die; q.w: quaque week; SD: standard deviation; t.i.d: ter in die; TNF-α: tumor necrosis factor alpha; U: unit; w: weeks; y: years.

**Table 2:** Methodological quality of the included studies according to the risk of bias 2.0.

First Author (Year)	D1	D2	D3	D4	D5	Overall
Zhou (2002)	L	Sc	L	Sc	Sc	Sc
Zhao (2003)	L	Sc	L	Sc	Sc	Sc
Chen (2004)	L	Sc	L	Sc	Sc	Sc
Lu (2005)	L	Sc	L	Sc	Sc	Sc
Liu (2005)	L	Sc	L	Sc	Sc	Sc
He (2005)	L	Sc	L	Sc	Sc	Sc
Qiu (2005)	L	Sc	L	Sc	Sc	Sc
Li (2006)	L	Sc	L	Sc	Sc	Sc
Li (2008)	L	Sc	L	Sc	Sc	Sc
Ye (2008)	L	Sc	L	Sc	Sc	Sc
Zhong (2008)	L	Sc	L	Sc	Sc	Sc
Hu (2009)	L	Sc	L	Sc	Sc	Sc
Wang (2009)	L	Sc	L	Sc	Sc	Sc
Feng (2009)	L	Sc	L	Sc	Sc	Sc
Xie (2009)	L	Sc	L	Sc	Sc	Sc
Hou (2009)	L	Sc	L	Sc	Sc	Sc
Ho (2010)	L	L	L	L	Sc	Sc
Si (2010)	L	Sc	L	Sc	Sc	Sc
Yan (2010)	L	Sc	L	Sc	Sc	Sc
Ma (2010)	L	Sc	L	Sc	Sc	Sc
Ma (2011)	L	Sc	L	Sc	Sc	Sc
Wang (2011)	L	Sc	L	Sc	Sc	Sc
Xie (2012)	L	Sc	L	Sc	Sc	Sc
Jia (2012)	L	Sc	L	Sc	Sc	Sc
Cheng (2012)	L	Sc	L	Sc	Sc	Sc
Ma (2012a)	L	Sc	L	Sc	Sc	Sc
Ma (2012b)	L	Sc	L	Sc	Sc	Sc
Zhang (2013)	L	Sc	L	Sc	Sc	Sc
Liu (2013)	L	Sc	L	Sc	Sc	Sc
Xu (2013a)	L	Sc	L	Sc	Sc	Sc
Xu (2013b)	L	Sc	L	Sc	Sc	Sc
Zhu (2014)	L	Sc	L	Sc	Sc	Sc
Chen (2014)	L	Sc	L	Sc	Sc	Sc
Qian (2014)	L	Sc	L	Sc	Sc	Sc
Peng (2014)	L	Sc	L	Sc	Sc	Sc
Dou (2014)	L	Sc	L	Sc	Sc	Sc
Miao (2014)	L	Sc	L	Sc	Sc	Sc
Xu (2015)	L	Sc	L	Sc	Sc	Sc
Zhang (2015)	L	Sc	L	Sc	Sc	Sc
Yang (2015)	L	Sc	L	Sc	Sc	Sc
Liang (2015)	L	Sc	L	Sc	Sc	Sc
Han (2015)	L	Sc	L	Sc	Sc	Sc
Xiang (2016)	L	Sc	L	Sc	Sc	Sc
Wang (2016)	L	Sc	L	Sc	Sc	Sc
Zhou (2016)	L	Sc	L	Sc	Sc	Sc
Du (2016a)	L	Sc	L	Sc	Sc	Sc
Du (2016b)	L	Sc	L	Sc	Sc	Sc
Mao (2017)	L	Sc	L	Sc	Sc	Sc
Xia (2018)	L	Sc	L	Sc	Sc	Sc
Li (2018)	L	Sc	L	Sc	Sc	Sc
Yang (2018)	L	Sc	L	Sc	Sc	Sc
Wang (2019)	L	Sc	L	Sc	Sc	Sc
Zhang (2019)	L	Sc	L	Sc	Sc	Sc
Jiang (2019)	L	Sc	L	Sc	Sc	Sc
Wen (2019)	L	Sc	L	Sc	Sc	Sc
Chen (2020)	L	Sc	L	Sc	Sc	Sc

D1–D5: The 5 domain criteria; D1: bias arising from the randomization process; D2: bias due to deviations from intended interventions; D3: bias due to missing outcome data; D4: bias in measurement of the outcome; D5: bias in selection of the reported results; H: high risk of bias; L: low risk of bias; Sc: some concerns.

**Table 3:** Subgroup analysis of the trials that compared EAHM with CM for PASI 60.

	k	Risk Ratio	95% CI	Heterogeneity (I^2^)	P_subgroup_
**Type of Comparator**					0.0059
**•** **Acitretin**	14	1.1158	1.0236 to 1.2163	69.5%	
**•** **Other conventional medicine**	15	1.3114	1.2154 to 1.4151	13.9%	

EAHM: East Asian herbal medicine; CM: conventional medicine; PASI: psoriasis area severity index.

**Table 4:** Summary of findings for studies meta-analysis.

Intervention and Comparator Intervention	Outcomes	Number of Participants (Studies)	Anticipated Absolute Effects (95% CI)	Quality of the Evidence (GRADE)
EAHM compared to CM for inflammatory skin manifestation of plaque psoriasis	PASI 70	1865 (18 trials)	161 more per 1000 (from 108 more to 218 more)	⊕⊕⊕◯ MODERATE ^a^
	PASI 60	2479 (29 trials)	126 more per 1000 (from 75 more to 182 more)	⊕⊕◯◯ LOW ^a,c^
	Continuous PASI score	2139 (27 trials)	MD 2.3386 point lower (3.3068 lower to 1.3704 lower)	⊕⊕◯◯ LOW ^a,c^
	IL-17	262 (4 trials)	SMD 1.17 SD lower (2.18 lower to 0.16 lower)	⊕⊕◯◯ LOW ^a,c^
	IL-23	262 (4 trials)	SMD 1.3204 SD lower (3.0143 lower to 0.3734 higher)	⊕◯◯◯ VERY LOW ^a,b,c^
	TNF-α	584 (5 trials)	SMD 1.4396 SD lower (2.8303 lower to 0.499 lower)	⊕⊕◯◯ LOW ^a,c^
	DLQI	259 (4 trials)	MD 3.1161 point lower (4.2796 lower to 1.9526 lower)	⊕⊕⊕◯ MODERATE ^a^

EAHM: East Asian herbal medicine; CM: conventional medicine; DLQI: Dermatology Life Quality Index; IL-17: Interlukin-17; IL-23: Interlukin-23 MD: mean difference; PASI: psoriasis area severity index; RCT: randomized clinical trial; RR: risk ratio; SD: standardized difference; SMD: standardized mean difference; TNF-α: tumor necrosis factor alpha. GRADE working group grades of evidence. High quality: Further research is very unlikely to change our confidence in the estimate of effect. Moderate quality: Further research is likely to have an important impact on our confidence in the estimate of effect and may change the estimate. Low quality: Further research is very likely to have an important impact on our confidence in the estimate of effect and is likely to change the estimate. Very low quality: very uncertain about the estimate. ^a^: Study design with some bias in randomized or distributed blind. ^b^: The 95% confidence interval passes 0 (MD and SMD) or 1 (RR and OR) and the other interventions (OIs) are not satisfied. ^c^: The confidence intervals are less overlapping, or the heterogeneity is high.

**Table 5:** The ingredients of EHAM used in the included studies.

First Author (Year)	EAHM Prescription Name	Source	Ingredients of EAHM Prescription (Latin Name)	Types of Preparation
Zhou (2002)	Yuyin capsule	Prepared by Zhou (2002)	Rehmanniae Radix Recens 20 g, Smilacis Rhizoma 20 g, Salviae Miltiorrhizae Radix 15 g, Sophorae Tonkinensis Radix Et Rhizoma 10 g, Paeoniae Radix Alba 10 g, Moutan Radicis Cortex 10 g, Manitis Squama 10 g, Zaocys 10 g, Hedyotidis Herba 10 g, Glycyrrhizae Radix et Rhizoma 5 g	Capsule
Zhao (2003)	Xiaoyin decoction	Prepared by Zhao (2003)	Sophorae Tonkinensis Radix Et Rhizoma 15 g, Scutellariae Barbatae Herba 15 g, Sargentodoxa Cuneata 20 g, Rhizoma Paridis 15 g, Smilax china Linn 30 g, Rehmanniae Radix Recens 30 g, Moutan Radicis Cortex 15 g, Sophorae Flos 15 g, Gentianae Macrophyllae Radix 30 g, Smilacis Rhizoma 20 g, Tripterygium wilfordii 20 g, Scorpio 5 g, Scolopendra 2 pieces, Vespae Nidus 20 g, Euonymi Lignum Suberalatum 20 g, Radix Paeoniae Rubra 15 g	Decoction
Chen (2004)	Sanlong Sanchong decoction	Shaanxi Yulin Chinese Pharmaceutical Co., Ltd.	Portulacae Herba, Smilacis Rhizoma, Dictamni Radicis Cortex, Angelicae Dahuricae Radix, Indigo Pulverata Levis, Lithospermi Radix, Salviae Miltiorrhizae Radix, Taraxaci Herba, Dryopteridis Crassirhizomatis Rhizoma, Tokoro Rhizoma, Mume Fructus, Schisandrae Fructus, Crataegi Fructus, Massa Medicata Fermentata	Capsule
Lu (2005)	Yinxieling capsule	Prepared by Lu (2005)	Bubali Cornu, Rehmanniae Radix Recens, Imperatae Rhizoma, Sophorae Flos, Lithospermi Radix, Angelicae Gigantis Radix, Salviae Miltiorrhizae Radix, Moutan Radicis Cortex, Smilacis Rhizoma, Dictamni Radicis Cortex, Hedyotidis Herba, Deinagkistrodon	Capsule
Liu (2005)	Jiedu Liangxue decoction	Prepared by Liu (2005)	Rehmanniae Radix Recens 30 g, Lithospermi Radix 15 g, Sophorae Flos 30 g, Isatidis Radix 15 g, Dictamni Radicis Cortex 15 g, Rhizoma Paridis 15 g, Salviae Miltiorrhizae Radix 15 g, Scrophulariae Radix 15 g, Glycyrrhizae Radix et Rhizoma 6 g	Decoction
He (2005)	Antidote decoction	Prepared by He (2005)	Sparganii Rhizoma 10 g, Curcumae Rhizoma 10 g, Scutellariae Radix 10 g, Smilacis Rhizoma 30 g, Hedyotidis Herba15 g, Lithospermi Radix 12 g, Taraxaci Herba 15 g, Mume Fructus 10 g	Decoction
Qiu (2005)	Sanyu Xiaoyin decoction	Prepared by Qiu (2005)	Sparganii Rhizoma 10 g, Curcumae Rhizoma 10 g, Persicae Semen 10 g, Carthami Flos 10 g, Spatholobi Caulis 10 g, Euonymi Lignum Suberalatum 30 g, Hedyotidis Herba 30 g, Salviae Miltiorrhizae Radix 30 g, Citri Unshius Pericarpium 30 g	Decoction
Li (2006)	EAHM prescription for individual clinical trial	Prepared by Li (2006)	Lithospermi Radix 15 g, Rubiae Radix 15 g, Isatidis Radix 30 g, Imperatae Rhizoma 30 g, Rehmanniae Radix Recens 15 g, Radix Paeoniae Rubra 15 g, Salviae Miltiorrhizae Radix 15 g, Hedyotidis Herba 15 g, Spatholobi Caulis 30 g, Smilacis Rhizoma 15 g, Sophorae Flos 15 g, Gazellae seu Saigae Cornu 0.6 g	Decoction
Li (2008)	Qinzhu Liangxue decoction	Prepared by Li (2008)	Magenetitum 30 g, Margaritifera Concha 25 g, Ostreae Testa 30 g, Scutellariae Radix 9 g, Lithospermi Radix 9 g, Cynanchi Paniculati Radix Et Rhizoma 9 g, Coicis Semen 10 g, Saposhnikoviae Radix 9 g, Glycyrrhizae Radix et Rhizoma 6 g	Decoction
Ye (2008)	Zhixue Jiedu Xiaoyin decoction	Prepared by Ye (2008)	Rehmanniae Radix Recens 10–20 g, Lithospermi Radix 10 g, Radix Paeoniae Rubra 10 g, Moutan Radicis Cortex 10 g, Angelicae Gigantis Radix 10 g, Cnidii Rhizoma 6–10 g, Carthami Flos 3–6 g, Flos Persicae 6–10 g, Zaocys 10–15 g, Tribuli Fructus 10 g, Lonicerae Flos 10–15 g, Smilacis Rhizoma 10–30 g	Decoction
Zhong (2008)	Xiaoyin granule	Epons pharmaceutical Co., Ltd.	Rehmanniae Radix Recens 10 g, Angelicae Gigantis Radix 10 g, Radix Paeoniae Rubra 10 g, Cnidii Rhizoma 6 g, Hedyotidis Herba 15 g, Lithospermi Radix 6 g, Curcumae Rhizoma 10 g, Smilacis Rhizoma 15 g, Mume Fructus 10 g, Scutellariae Barbatae Herba 15 g, Glycyrrhizae Radix et Rhizoma 3 g	Decoction
Hu (2009)	Liangxue decoction	Prepared by Hu (2009)	Bubali Cornu 30 g, Notoginseng Radix et Rhizoma 3 g, Lithospermi Radix 10 g, Coptidis Rhizoma 3 g, Scutellariae Radix 10 g, Phellodendri Cortex 15 g, Coicis Semen 15 g, Poria Sclerotium 15 g, Glycyrrhizae Radix et Rhizoma 5 g	Decoction
Hou (2009)	Huoxueliangxue decoction	Prepared by Hou (2009)	Sophorae Flos 30 g, Imperatae Rhizoma 30 g, Lithospermi Radix 15 g, Moutan Radicis Cortex 15 g, Rubiae Radix 15 g, Rehmanniae Radix Recens 30 g, Salviae Miltiorrhizae Radix 15 g, Spatholobi Caulis 30 g, Isatidis Radix 30 g, Dictamni Radicis Cortex 15 g	Decoction
Wang (2009)	Baiji decoction	Prepared by Wang (2009)	Rehmanniae Radix Recens 30 g, Moutan Radicis Cortex 15 g, Isatidis Folium 30 g, Imperatae Rhizoma 30 g, Hedyotidis Herba 20 g, Salviae Miltiorrhizae Radix 30 g, Cicadidae Periostracum 15 g, Batryticatus Bombyx 15 g, Zaocys 15 g, Astragali Radix 30 g, Glycyrrhizae Radix et Rhizoma 10 g	Decoction
Feng (2009)	Wushe decoction	Prepared by Fang (2009)	Zaocys 20 g, Kalopanacis Cortex 15 g, Radix Paeoniae Rubra 10 g, Saposhnikoviae Radix 10 g, Ecliptae Herba 10 g, Rehmanniae Radix Recens 10 g, Dictamni Radicis Cortex 15 g, Junci Medulla 6 g, Smilacis Rhizoma 15 g, Spatholobi Caulis 15 g, Persicae Semen 10 g, Lithospermi Radix 10 g, Glycyrrhizae Radix et Rhizoma 6 g	Decoction
Xie (2009)	Kangyin 1 decoction	Prepared by Xie (2009)	Rehmanniae Radix Recens 30 g, Hedyotidis Herba 30 g, Smilacis Rhizoma 30 g, Dictamni Radicis Cortex 20 g, Salviae Miltiorrhizae Radix 15 g, Isatidis Folium 15 g, Sophorae Flos 15 g, Polygoni Multiflori Caulis 15 g, Moutan Radicis Cortex 12 g, Radix Paeoniae Rubra 12 g, Lithospermi Radix 12 g Sophorae Tonkinensis Radix Et Rhizoma 6 g, Glycyrrhizae Radix et Rhizoma 6 g	Decoction
Ho (2010)	Wen-tong-hua-yu formulation	Prepared by Ho (2010)	Ephedrae Herba 6 g, Aconiti Lateralis Radix Preparata 10 g, Sinapis Semen 10 g, Cinnamomi Cortex 3 g, Zingiberis Rhizoma 3 g, Cornu Cervi Degelatinatum 15 g, Rehmanniae Radix Preparata 10 g, Smilacis Rhizoma 60 g, Dictamni Radicis Cortex 30 g, Imperatae Rhizoma 30 g, Salviae Miltiorrhizae Radix 15 g, Spatholobi Caulis 30 g, Lithospermi Radix 30 g, Sophorae Flos 30 g, Glycyrrhizae Radix et Rhizoma 6 g, Indigo Pulverata Levis 6 g	Decoction
Si (2010)	Jiawei Xiaoyaosan	Prepared by Si (2010)	Moutan Radicis Cortex 10 g, Gardeniae Fructus 10 g, Bupleuri Radix 6 g, Angelicae Gigantis Radix 10 g, Paeoniae Radix Alba 10 g, Poria Sclerotium 12 g, Atractylodis Rhizoma Alba 10 g, Menthae Herba 6 g, Zingiberis Rhizoma Recens 3 g, Glycyrrhizae Radix et Rhizoma 6 g	Decoction
Yan (2010)	Quyin decoction	Prepared by Yan (2010)	Rehmanniae Radix Recens, Rehmanniae Radix Preparata, Angelicae Gigantis Radix, Persicae Semen, Lithospermi Radix, Salviae Miltiorrhizae Radix, Carthami Flos, Cremastrae Tuber	Decoction
Ma (2010)	Psoriasis prescription	Prepared by Ma (2010)	Eupolyphaga 10 g, Indigo Pulverata Levis 10 g, Glycyrrhizae Radix et Rhizoma 10 g, Salviae Miltiorrhizae Radix 30 g, Hedyotidis Herba 30 g, Rehmanniae Radix Recens 30 g	Decoction
Ma (2011)	Keyin I prescription	Prepared by Ma (2011)	Lithospermi Radix 15 g, Radix Paeoniae Rubra 12 g, Rehmanniae Radix Recens 15 g, Carthami Flos 12 g, Angelicae Gigantis Radix 12 g, Scorpio 6 g, Bubali Cornu 20 g, Scolopendra 2 pieces, Sargentodoxa Cuneata 30 g, Scutellariae Radix 12 g, Forsythiae Fructus 12 g	Decoction
Wang (2011)	Tufuling Qingdai decoction	Prepared by Wang (2011)	Smilacis Rhizoma 30 g, Indigo Pulverata Levis 6 g, Lonicerae Flos 20 g, Glycyrrhizae Radix et Rhizoma 6 g, Tribuli Fructus 30 g, Sophorae Tonkinensis Radix Et Rhizoma 10 g, Dryopteridis Crassirhizomatis Rhizoma 15 g, Euphorbiae Humifusae Herba 30 g, Scorpio 3 g, Scolopendra 2 pieces, Lycii Radicis Cortex 15 g, Moutan Radicis Cortex 10 g	Decoction
Xie (2012)	Liangxie Runfu decoction	Prepared by Xie (2012)	Rehmanniae Radix Recens 30 g, Isatidis Folium 30 g, Smilacis Rhizoma 30 g, Imperatae Rhizoma 12 g, Sophorae Flos 15 g, Moutan Radicis Cortex 15 g, Glycyrrhizae Radix et Rhizoma 6 g	Decoction
Jia (2012)	Xiaobi decoction	Prepared by Jia (2012)	Lonicerae Flos 15 g, Forsythiae Fructus 15 g, Violae Herba 15 g, Taraxaci Herba 15 g, Rehmanniae Radix Recens 20 g, Zaocys 15 g, Vespae Nidus 10 g, Scutellariae Radix 15 g, Carthami Flos 15 g, Hirudo 10 g, Pinelliae Tuber 15 g	Decoction
Cheng (2012)	EAHM prescription for individual clinical trial	Prepared by Cheng (2012)	Bubali Cornu 10–15 g, Smilacis Rhizoma 6–10 g, Imperatae Rhizoma 10–15 g, Scutellariae Radix 6–10 g, Taraxaci Herba 10–15 g, Moutan Radicis Cortex 6–10 g, Kochiae Fructus 6 g, Spatholobi Caulis 6 g, Isatidis Radix 10 g	Decoction
Ma (2012a)	Liangxue jiedu decoction	Prepared by Ma (2012a)	Sophorae Flos 30 g, Imperatae Rhizoma 30 g, Lithospermi Radix 15 g, Radix Paeoniae Rubra 15 g, Rehmanniae Radix Recens 15 g, Moutan Radicis Cortex 15 g, Salviae Miltiorrhizae Radix 15 g, Isatidis Radix 30 g, Isatidis Folium 30 g, Lonicerae Flos 15 g, Forsythiae Fructus 12 g, Dictamni Radicis Cortex 15 g	Decoction
Ma (2012b)	Yinxiaobing decoction	Prepared by Ma (2012b)	Eupolyphaga 10 g, Salviae Miltiorrhizae Radix 30 g, Hedyotidis Herba 30 g, Indigo Pulverata Levis 10 g, Rehmanniae Radix Recens 30 g, Glycyrrhizae Radix et Rhizoma 10 g	Decoction
Zhang (2013)	Blood-cooling decoction	Prepared by Zhang (2013)	Rehmanniae Radix Recens 20 g, Moutan Radicis Cortex 15 g, Radix Paeoniae Rubra 15 g, Scrophulariae Radix 10 g, Sophorae Flos 10 g, Dictamni Radicis Cortex 10 g, Forsythiae Fructus 10 g, Lonicerae Flos 10 g, Smilacis Rhizoma 10 g, Saposhnikoviae Radix 10 g, Cicadidae Periostracum 10 g, Glycyrrhizae Radix et Rhizoma 10 g	Decoction
Liu (2013)	Wanji decoction	Prepared by Liu (2013)	Rehmanniae Radix Recens 20 g, Moutan Radicis Cortex 10 g, Dictamni Radicis Cortex 20 g, Hedyotidis Herba 20 g, Rhizoma Paridis 15 g, Schizonepetae Spica 10 g, Saposhnikoviae Radix 10 g, Mori Radicis Cortex 20 g, Scutellariae Radix 15 g, Lonicerae Flos 20 g, Taraxaci Herba 20 g, Forsythiae Fructus 20 g, Isatidis Radix 20 g, Isatidis Folium 10 g, Glycyrrhizae Radix et Rhizoma 10 g	Decoction
Xu (2013a)	Shufeng Yangxue decoction	Prepared by Xu (2013a)	Rehmanniae Radix Recens 18 g, Radix Paeoniae Rubra 15 g, Angelicae Gigantis Radix 15 g, Cnidii Rhizoma 10 g, Isatidis Radix 20 g, Lithospermi Radix 10 g, Cnidi Fructus 18 g, Sophorae Radix 18 g, Dictamni Radicis Cortex 15 g, Glycyrrhizae Radix et Rhizoma 6 g	Decoction
Xu (2013b)	Qingre Liangxue decoction	Prepared by Xu (2013b)	Bubali Cornu 15 g, Flos Sophorae Immaturus 12 g, Imperatae Rhizoma 30 g, Scutellariae Radix 30 g, Rehmanniae Radix Recens 30 g, Radix Paeoniae Rubra 12 g, Moutan Radicis Cortex 12 g, Spatholobi Caulis 30 g, Campsitis Flos 12 g, Salviae Miltiorrhizae Radix 30 g, Isatidis Radix 30 g	Decoction
Zhu (2014)	Dahuang Zhechong Capsule	Jiangsu Ehai Pharmaceutical Co., Ltd.	Rhei Radix et Rhizoma, Eupolyphaga, Persicae Semen, Lacca Rhois Exsiccata, Hirudo, Tabanus, Holotrichia, Scutellariae Radix, Persicae Semen, Armeniacae Semen, Rehmanniae Radix Preparata, Glycyrrhizae Radix et Rhizoma, Paeoniae Radix Alba	Capsule
Chen (2014)	Liangxue No.1 formula	Prepared by Chen (2014)	Bubali Cornu 20 g, Angelicae Gigantis Radix 10 g, Rehmanniae Radix Recens 15 g, Saposhnikoviae Radix 10 g, Cicadidae Periostracum 6 g, Anemarrhenae Rhizoma 6 g, Sophorae Radix 6 g, Sesami Semen Nigra 6 g, Schizonepetae Spica 10 g, Atractylodis Rhizoma 6 g, Arctii Fructus 6 g, Gypsum Fibrosum 10 g, Glycyrrhizae Radix et Rhizoma 3 g, Akebiae Caulis 3 g, Moutan Radicis Cortex 10 g, Radix Paeoniae Rubra 10 g	Decoction
Qian (2014)	Liangxue Runfu decoction	Prepared by Qian (2014)	Radix Paeoniae Rubra 12 g, Dictamni Radicis Cortex 12 g, Salviae Miltiorrhizae Radix 12 g, Saposhnikoviae Radix 15 g, Tribuli Fructus 15 g, Smilacis Rhizoma 30 g, Isatidis Folium 30 g, Rehmanniae Radix Recens 30 g, Sophorae Flos 15 g, Solani Nigri Herba 15 g, Moutan Radicis Cortex 15 g, Imperatae Rhizoma 12 g, Glycyrrhizae Radix et Rhizoma 6 g	Decoction
Peng (2014)	Liangxue Jiedu decoction	Prepared by Peng (2014)	Sophorae Flos 30 g, Isatidis Radix 30 g, Smilacis Rhizoma 30 g, Isatidis Folium 30 g, Imperatae Rhizoma 15~30 g, Sophorae Tonkinensis Radix Et Rhizoma 15 g, Lithospermi Radix 15 g, Rehmanniae Radix Recens 15 g, Moutan Radicis Cortex 15 g, Salviae Miltiorrhizae Radix 15 g, Lonicerae Flos 15 g, Dictamni Radicis Cortex 15 g, Forsythiae Fructus 12 g	Decoction
Dou (2014)	Wuteng Xiaoyin decoction	Prepared by Dou (2014)	Zaocys 15~30 g, Sargentodoxa Cuneata 20 g, Polygoni Multiflori Radix 20 g, Spatholobi Caulis 20 g, Curcumae Radix 15 g, Salviae Miltiorrhizae Radix 30 g, Radix Paeoniae Rubra 15 g, Rehmanniae Radix Recens 20 g, Persicae Semen 10 g, Rhei Radix et Rhizoma 3 g, Smilacis Rhizoma 30 g, Dictamni Radicis Cortex 20 g, Glycyrrhizae Radix et Rhizoma 6 g	Decoction
Miao (2014)	Quyin decoction	Prepared by Miao (2014)	Tripterygium wilfordii 50 g, Hedyotidis Herba 50 g, Astragali Radix 30 g, Salviae Miltiorrhizae Radix 30 g	Decoction
Xu (2015)	Liangxue Jiedu decoction	Prepared by Xu (2015)	Bubali Cornu 30 g, Rehmanniae Radix Recens 30 g, Moutan Radicis Cortex 10 g, Radix Paeoniae Rubra 10 g, Angelicae Gigantis Radix 12 g, Cnidii Rhizoma 10 g, Schizonepetae Spica 10 g, Saposhnikoviae Radix 10 g, Hedyotidis Herba 15 g, Lonicerae Flos 10 g, Salviae Miltiorrhizae Radix 15 g, Spatholobi Caulis 20 g, Lithospermi Radix 15 g, Dictamni Radicis Cortex 15 g, Glycyrrhizae Radix et Rhizoma 10 g	Decoction
Zhang (2015)	Zinyin Qingre Xiaofeng san	Prepared by Zhang (2015)	Rehmanniae Radix Recens 30 g, Moutan Radicis Cortex 10 g, Radix Paeoniae Rubra 10 g, Liriopis seu Ophiopogonis Tuber 10 g, Scrophulariae Radix 10 g, Salviae Miltiorrhizae Radix 10 g, Cannabis Semen 10 g, Isatidis Folium 10 g, Sophorae Tonkinensis Radix Et Rhizoma 10 g, Dictamni Radicis Cortex 10 g	Decoction
Yang (2015)	Qingre Liangxue decoction	Prepared by Yang (2015)	Bubali Cornu 15 g, Radix Paeoniae Rubra 12 g, Flos Sophorae Immaturus 12 g, Moutan Radicis Cortex 12 g, Campsitis Flos 12 g, Scutellariae Radix 30 g, Isatidis Radix 30 g, Rehmanniae Radix Recens 30 g, Spatholobi Caulis 30 g, Imperatae Rhizoma 30 g, Salviae Miltiorrhizae Radix 30 g	Decoction
Liang (2015)	Tufuling Qingdai decoction	Prepared by Liang (2015)	Smilacis Rhizoma 30 g, Indigo Pulverata Levis 6 g, Lonicerae Flos 20 g, Glycyrrhizae Radix et Rhizoma 6 g, Tribuli Fructus 30 g, Sophorae Tonkinensis Radix Et Rhizoma 10 g, Dryopteridis Crassirhizomatis Rhizoma 15 g, Lithospermi Radix 20 g, Euphorbiae Humifusae Herba 30 g, Scorpio 3 g, Scolopendra 2 pieces, Moutan Radicis Cortex 10 g	Decoction
Han (2015)	Huayinjiedu decoction	Prepared by Han (2015)	Rehmanniae Radix Recens, Radix Paeoniae Rubra, Salviae Miltiorrhizae Radix, Lithospermi Radix, Imperatae Rhizoma, Lonicerae Flos, Dioscorea bulbifera Rhizoma, Tribuli Fructus, Smilacis Rhizoma	Decoction
Xiang (2016)	Qinzhu Liangxue decoction	Prepared by Xiang (2016)	Scutellariae Radix 12 g, Margaritifera Concha 12 g, Salviae Miltiorrhizae Radix 15 g, Lithospermi Radix 9 g, Fluoritum 30 g,	Decoction
Wang (2016)	Liangxue Runfu decoction	Prepared by Wang (2006)	Smilacis Rhizoma 30 g, Isatidis Folium 30 g, Rehmanniae Radix Recens 30 g, Radix Paeoniae Rubra 12 g, Dictamni Radicis Cortex 12 g, Salviae Miltiorrhizae Radix 12 g, Saposhnikoviae Radix 15 g, Tribuli Fructus 15 g, Sophorae Flos 12 g, Solani Nigri Herba 12 g, Moutan Radicis Cortex 12 g, Imperatae Rhizoma 12 g, Glycyrrhizae Radix et Rhizoma 6 g.	Decoction
Zhou (2016)	Shufeng jiedu capsules	Anhui Jiren Pharmaceutical Industry Co., Ltd.	Isatidis Radix, Polygoni Cuspidati Rhizoma et Radix, Forsythiae Fructus, Patriniae Radix, Bupleuri Radix, Phragmitis Rhizoma, Verbenae Herba, Glycyrrhizae Radix et Rhizoma	Capsule
Du (2016a)	Shengdi Baimao decoction	Prepared by Du (2016a)	Rehmanniae Radix Recens 20 g, Imperatae Rhizoma 20 g, Smilacis Rhizoma 20 g, Poria Sclerotium 20 g, Coicis Semen 20 g, Sophorae Flos 15 g, Lithospermi Radix 10 g, Spatholobi Caulis 10 g, Atractylodis Rhizoma 10 g	Decoction
Du (2016b)	Heat-clearing and detoxicating oral liquid	Beijing Tongrentang Technology Development Co., Ltd.	Gypsum Fibrosum, Lonicerae Flos, Scrophulariae Radix, Rehmanniae Radix Recens, Forsythiae Fructus, Gardeniae Fructus, Gueldenstaedtia Verna, Gentianae Scabrae Radix et Rhizoma, Isatidis Radix, Anemarrhenae Rhizoma, Liriopis seu Ophiopogonis Tuber	Liquid
Mao (2017)	Xiaoyinfang	Prepared by Mao (2017)	Sophorae Tonkinensis Radix Et Rhizoma, Imperatae Rhizoma, Isatidis Radix, Sophorae Radix, Euphorbiae Helioscopiae Herba, Lithospermi Radix, Rhei Radix et Rhizoma, Rehmanniae Radix Recens, Scutellariae Radix, Salviae Miltiorrhizae Radix, Sargentodoxa Cuneata, Dictamni Radicis Cortex	Deccotion
Xia (2018)	Kanli fang	Prepared by Xia (2018)	Anemarrhenae Rhizoma 10 g, Rehmanniae Radix Recens 10 g, Glehniae Radix 10 g, Liriopis seu Ophiopogonis Tuber 10 g, Gardeniae Fructus 10 g, Lilii Bulbus 10 g, Lophatheri Herba 10 g, Zizyphi Semen 10 g, Phellodendri Cortex 10 g, Corni Fructus 9 g, Prunellae Spica 10 g	Decoction
Li (2018)	Jianpi Jiedu decoction	Prepared by Li (2018)	Smilacis Rhizoma 30 g, Poria Sclerotium 12 g, Atractylodis Rhizoma Alba 10 g, Tokoro Rhizoma 10 g, Sophorae Radix 10 g, Hedyotidis Herba 30 g, Forsythiae Fructus 15 g, Salviae Miltiorrhizae Radix 10 g, Phellodendri Cortex 10 g, Coicis Semen 30 g	Decoction
Yang (2018)	Jinyu Xiaoyin granules	Shaanxi Kanghui Pharmaceutical Co., Ltd.	Curcumae Radix, Tribuli Fructus, Angelicae Gigantis Radix, Curcumae Rhizoma, Sargentodoxa Cuneata, Clematidis Radix, Paeoniae Radix Alba, Dictamni Radicis Cortex, Cnidi Fructus	Granule
Wang (2019)	Qingying tang	Prepared by Wang (2019)	Gazellae seu Saigae Cornu 0.6 g, Rehmanniae Radix Recens 15 g, Moutan Radicis Cortex 12 g, Forsythiae Fructus 15 g, Isatidis Radix 15 g, Imperatae Rhizoma 15 g, Radix Paeoniae Rubra 10 g, Plantaginis Semen 15 g, Spatholobi Caulis 10 g, Lonicerae Flos 20 g, Taraxaci Herba 10 g	Decoction
Zhang (2019)	Xijiao Dihuang decoction	Prepared by Zhang (2019)	Bubali Cornu 30 g, Rehmanniae Radix Recens 24 g, Forsythiae Fructus 15 g, Lonicerae Flos 15 g, Paeoniae Radix Alba 12 g, Moutan Radicis Cortex 9 g, Platycodonis Radix 6 g, Menthae Herba 6 g, Arctii Fructus 6 g, Glycyrrhizae Radix et Rhizoma 5 g, Schizonepetae Spica 5 g, Glycine Semen Preparata 6 g, Lophatheri Herba 4 g	Decoction
Jiang (2019)	Xijiao Dihuang Jiedu decoction	Prepared by Jiang (2019)	Bubali Cornu 30 g, Zingiberis Rhizoma Recens 20 g, Moutan Radicis Cortex 20 g, Radix Paeoniae Rubra 20 g	Decoction
Wen (2019)	Xiaobi decoction	Prepared by Wen (2019)	Lonicerae Flos 15 g, Forsythiae Fructus 15 g, Scutellariae Radix 15 g, Rehmanniae Radix Recens 20 g, Zaocys 15 g, Violae Herba 15 g, Pinelliae Tuber 15 g, Persicae Semen 15 g, Carthami Flos 15 g, Taraxaci Herba 15 g, Vespae Nidus 10 g, Hirudo 10 g, Cinnamomi Ramulus 8 g	Decoction
Chen (2020)	Quyin decoction	Prepared by Chen (2020)	Glycyrrhizae Radix et Rhizoma 5 g, Smilacis Rhizoma 30 g, Moutan Radicis Cortex 10 g, Bubali Cornu 15 g, Rehmanniae Radix Recens 15 g, Isatidis Radix 15 g, Dictamni Radicis Cortex 10 g, Scrophulariae Radix 15 g, Kochiae Fructus 15 g, Hedyotidis Herba 30 g, Plantaginis Herba 10 g, Salviae Miltiorrhizae Radix 15 g, Alismatis Rhizoma 10 g	Decoction

EAHM: East Asian herbal medicine; NR: Not Reported; The Latin names of medicinal herbs were prepared based on “The Korean Pharmacopeia (KP)” and “The Korean Herbal Pharmacopeia (KHP)”.

**Table 6:** Characters of top 16 commonly prescribed herbs utilized with relative frequencies exceeding 20% inclusion trials.

No.	EAHM (Latin Name)	Frequency of Prescription	Relative Frequency (%)	Properties	Flavors	Action Category
1	Rehmanniae Radix Recens	39	69.64	Cold	Sweet	Clearing heat to cool blood
2	Salviae Miltiorrhizae Radix	28	50.00	Cold	Bitter	Activating blood and removing blood stasis
3	Glycyrrhizae Radix et Rhizoma	27	48.21	Neutral	Sweet	Tonifying qi
4	Moutan Radicis Cortex	27	48.21	Cold	Bitter	Clearing heat to cool blood
5	Lithospermi Radix	24	42.86	Cold	Sweet	Clearing heat to cool blood
6	Smilacis Rhizoma	24	42.86	Neutral	Sweet	Clearing heat and toxic materials
7	Radix Paeoniae Rubra	21	37.50	Cold	Bitter	Clearing heat to cool blood
8	Dictamni Radicis Cortex	20	35.71	Cold	Bitter	Clearing heat and dampness
9	Imperatae Rhizoma	17	30.36	Cold	Sweet	Cooling blood to arrest bleeding
10	Hedyotidis Herba	15	26.79	Cold	Bitter	Clearing heat and toxic materials
11	Isatidis Radix	15	26.79	Cold	Bitter	Clearing heat and toxic materials
12	Lonicerae Flos	14	25.00	Cold	Sweet	Clearing heat and toxic materials
13	Sophorae Flos	14	25.00	Cold	Bitter	Cooling blood to arrest bleeding
14	Scutellariae Radix	13	23.21	Cold	Bitter	Clearing heat and dampness
15	Forsythiae Fructus	12	21.43	Cold	Bitter	Clearing heat and toxic materials
16	Spatholobi Caulis	12	21.43	Warm	Bitter	Activating blood and removing blood stasis

EAHM: East Asian herbal medicine.

**Table 7:** Potential mechanism of core herbs included in this review.

First Author (Year)	EAHM (Latin Name)	Target Cell Line or Animal Model	Possible Active Ingredients	Possible Mechanisms
Sui (2013) [109]	Rehmanniae Radix Recens	-UVB ray treated mice	Radix Rehmanniae polysaccharides	-Enhancing serum IL-2, IL-4, and IL-10 levels -Enhancing skin GSH, SOD, CAT, and GSH-Px activities -Decreasing skin MDA level
Ma (2016) [110]	Salviae Miltiorrhizae Radix	-lipopolysaccharide-stimulated THP-1 macrophages	Tanshinone IIB, Danshixinkun B, Danshenol A, Arucadiol, Tanshindiol C, Salviolone, and Sugiol	-Inhibiting the expression of TNF-α, IL-1β, and IL-8
Yu (2017) [111]	Glycyrrhizae Radix et Rhizoma	-human monocyte model THP-1 -AD-like skin lesion model mice	Isoliquiritigenin	-Suppressing the up-regulation of CD86 and CD54 and abolished the DNCB-induced p38-α and ERK activation -Suppressing the DNCB-induced IgE and Th2 cytokines up-regulation -Inhibiting DNCB-induced pro-inflammatory cytokines such as TNF-α, IL-6 as well as IL-4 expressions
Yun (2013) [112]	Moutan Radicis Cortex	-stimulated with LPS in cultured HGFs	Paeonol, Paeoniflorin	-Inhibiting a wide variety of activations of inflammation-related genes
Kim (2007) [113]	Lithospermi Radix	-rat peritoneal mast cells -PCA rat	Shikonin	-Inhibiting the release of histamine in a dose-dependent manner -Inhibiting the anti-DNP IgE-induced passive cutaneous anaphylaxis reaction and IL-6, IL-8, and TNF-α expression -Inhibiting NF-κB activation and IκB-alpha degradation
Ki (2016) [114]	Smilacis Rhizoma	-AD-like skin lesion model mice	Astilbin, Neoastilbin, Isoastilbin, Neoisoastilbin, Engeletin and Isoengeletin	-Decreasing in both Th2 and Th1 serum antibodies -Suppressing expression of IL-4, IL-13, IL-17, IL-18, TSLP, and IFN-γ genes
Zhao (2016) [115]	Radix Paeoniae Rubra	-IMQ-induced psoriasis mice	Paeoniflorin	-Inhibiting IMQ-induced psoriasis by regulating Th17 cell response and cytokine secretion via phosphorylation of Stat3.
Yang (2017) [116]	Dictamni Radicis Cortex	-DNFB-induced CD mice	Fraxinellone	-Reducing the levels of TNF-α, IFN-γ, and IL-6 in inflamed tissues -Inhibiting enlargement of dorsal skin and prevented epidermal hyperplasia, hyperkeratosis, and spongiotic changes in inflamed tissues -Ameliorating skin lesions such as crust, scales, incrustation and petechiae, and lowered erythema index on skin surface
Ruan (2022) [117]	Imperatae Rhizoma	-LPS stimulated RAW 264.7 cells	Imperphenoside A, Imperphenols B and C, Imperphenosides D-F, Imperlignanosides A-D	-Nitric oxide inhibitory effects -Restraining the phosphorylation of nuclear factor kappa-B kinase to down-regulate the protein expression of inflammatory cytokines such as inducible nitric oxide synthase, interleukin-6 and tumor necrosis factor-α
Chen (2016) [118]	Hedyotidis Herba	-LPS stimulated RAW 264.7 cells	Total flavonoids	-Inhibiting the LPS-induced activation of NF-κB via the suppression of inhibitor of κB (IκB) phosphorylation -Reducing the phosphorylation of MAPK signaling molecules, which resulted in the inhibition of cytokine expression
Fan (2021) [119]	Isatidis Radix	-LPS stimulated RAW 264.7 cells	Acidic fraction	-Inhibiting the secretion of inflammatory cytokines (PGE2, IL-6, IL-1β, and NO, other than TNF-α) in a dose-dependent manner -Downregulating the expression of iNOS and COX-2 -Suppressing the phosphorylation of ERK1/2, JNK, and p38 -Reducing the translocation of NF-κB from the cytoplasm to nucleus
Wu (2020) [120]	Lonicerae Flos	-TPA (12-O-tetradecanoylphorbol-13-acetate)-induced ear edema mouse model -LPS-stimulated RAW264.7 cells	Chrysoeriol	-Lowering protein levels of phospho-p65 (Ser536), phospho-STAT3 (Tyr705), iNOS, COX-2, IL-6, IL-1β, and TNF-α -Decreasing the production of NO and PGE2 -Inhibiting the phosphorylation of inhibitor of κB (Ser32), p65 (Ser536), and Janus kinase 2 (Tyr1007/1008) -Decreasing nuclear localization of p50, p65, and STAT3 -Down regulating mRNA levels of pro-inflammatory cytokines IL-6, IL-1β and TNF-α
Lee (2013) [121]	Sophorae Flos	-BALB/c mice	Sophoricoside	-Inhibiting the phosphorylation and degradation of IκBα/β and the nuclear translocation of NF-κB p65 in B cells -Ameliorating DNCB-induced acute and chronic contact dermatitis
Wang (2022) [122]	Scutellariae Radix	- BALB/c mice treated with DNCB to induce AD-like skin lesions	Baicalin	-Up-regulating the protein expressions of filaggrin, involucrin, and loricrin -Inhibited the inflammatory response and the activation of NF-κB and JAK/STAT pathways -Inhibiting the release of IgE, histamine, TNF-α and IL-4
Sung (2016) [123]	Forsythiae Fructus	-Dermatophagoides farinae-induced atopic dermatitis in NC/Nga mice	Forsythoside A, Phillyrin, Pinoresinol, Phylligenin	-Attenuating serum levels of IgE, TNF-α, and histamine -Inhibiting the expression of chemokines, cytokines, and adhesion molecules -Inhibiting the production of chemokines in TNF-α/IFN-γ-activated human keratinocytes.
Tang (2020) [124]	Spatholobi Caulis	-cell model of oxygen-glucose deprivation	Spatholobi Caulis total extract	-Decreasing the protein expression of tissue factor -Enhancing SIRT1 protein expression and reduced Ace-p65 nuclear protein expression -Promotin protein expressions of nuclear Nrf2 and total HO-1

AD: atopic dermatitis; BALB/c: Bagg And Albino/c; CAT: catalase; CD54: intercellular adhesion molecule 1; CD86: cluster of differentiation 86; COX-2: cyclooxygenase-2; DNCB: 2,4-dinitrochlorobenzene; ERK: extracellular signal-regulated kinase; GSH: glutathione; GSH-Px: glutathione peroxidase; HGFs: human gingival fibroblasts; HO-1: heme oxygenase-1; IFN-γ: interferon-γ; IL: interleukin; IMQ: imiquimod; iNOS: inducible nitric oxide synthases; JAK/STAT: Janus kinase/signal transducer and activator of transcription; LPS: lipopolysaccharide; MDA: malondialdehyde; NF-κB: nuclear factor-κB; Nrf2: nuclear factor-erythroid 2 related factor 2; PCA: Passive cutaneous anaphylaxis; PGE2: prostaglandin E2; SIRT1: sirtuin 1; SOD: superoxide dismutase; Th1: T helper cell 1; Th2: T helper cell 2; TNF-α: tumor necrosis factor-α; TSLP: thymic stromal lymphopoietin; UVB; ultraviolet B.

## Data Availability

Data sharing is not applicable.

## Supplementary Materials

The following supporting information can be downloaded at: https://www.mdpi.com/article/10.3390/nu14122434/s1, Supplementary Table S1: PRISMA checklist; Supplementary Table S2: Search terms used in each database; Supplementary Figure S1. Bubble plot of PASI 60 for source of investigational medicine; Supplementary Figure S2. Bubble plot of PASI 60 for sample size; Supplementary Figure S3. Bubble plot of continuous PASI score for type of comparator; Supplementary Figure S4. Bubble plot of continuous PASI score for source of investigational medicine; Supplementary Figure S5. Bubble plot of continuous PASI score for sample size.
