# Supplementary material for: Oral Administration of East Asian Herbal Medicine for Inflammatory Skin Lesions in Plaque Psoriasis: A Systematic Review, Meta-Analysis, and Exploration of Core Herbal Materials
Source: Nutrients. 2022 Jun 12;14(12):2434. doi: 10.3390/nu14122434 (PMC9230602; doi:10.3390/nu14122434)
Supplement: Supplementary file 1 [file nutrients-14-02434-s001.zip › Supplementary Table S2 and Figures.pdf]

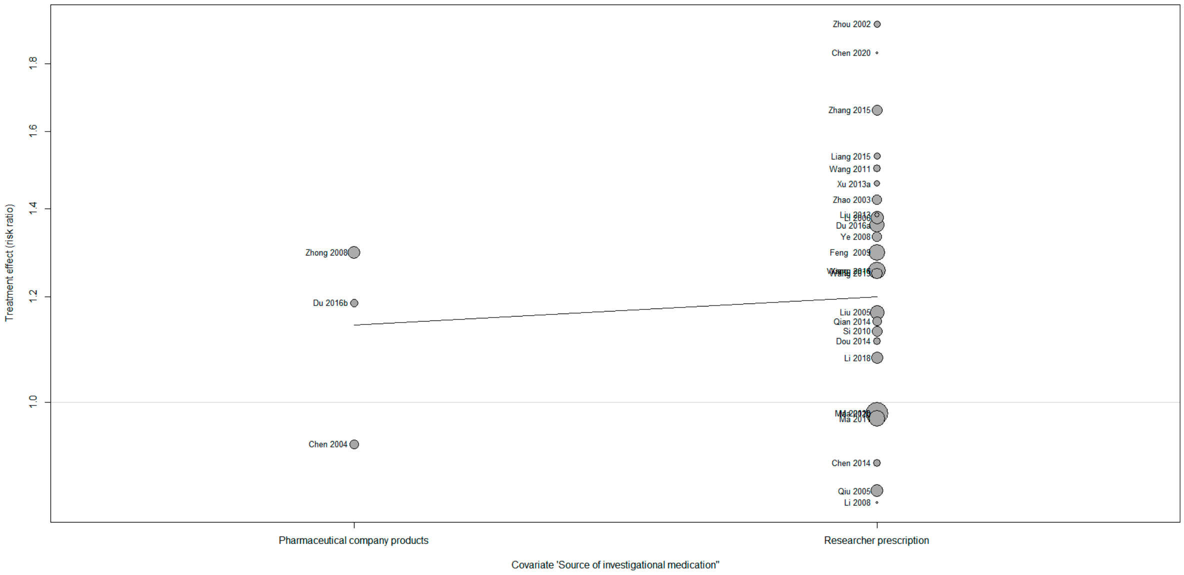

Supplementary Figure S1. Bubble plot of PASI 60 for source of investigational medicine.

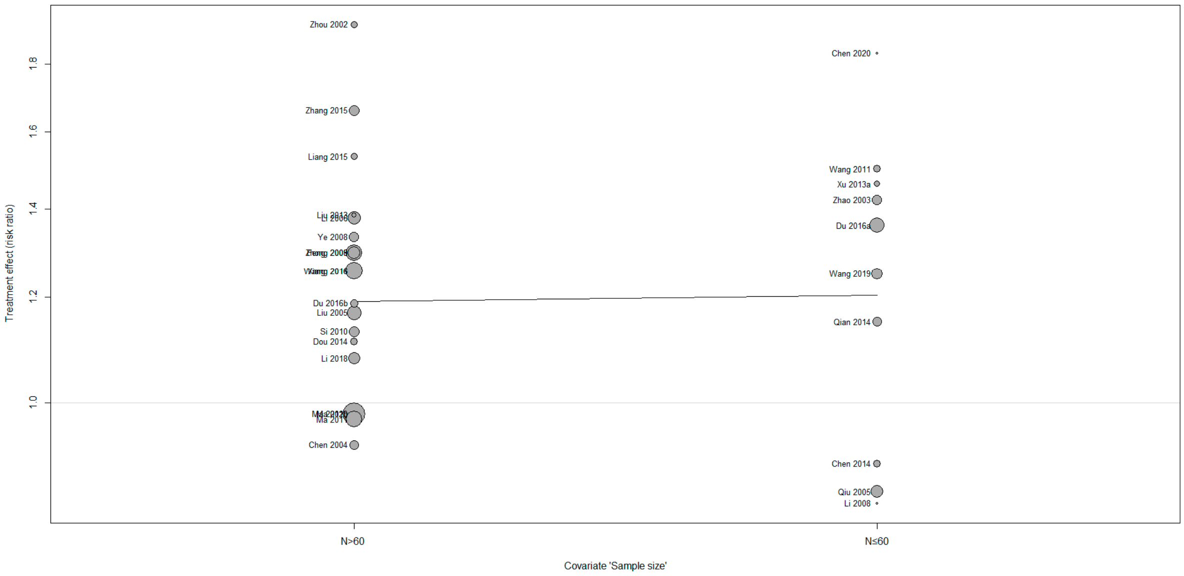

Supplementary Figure S2. Bubble plot of PASI 60 for sample size.

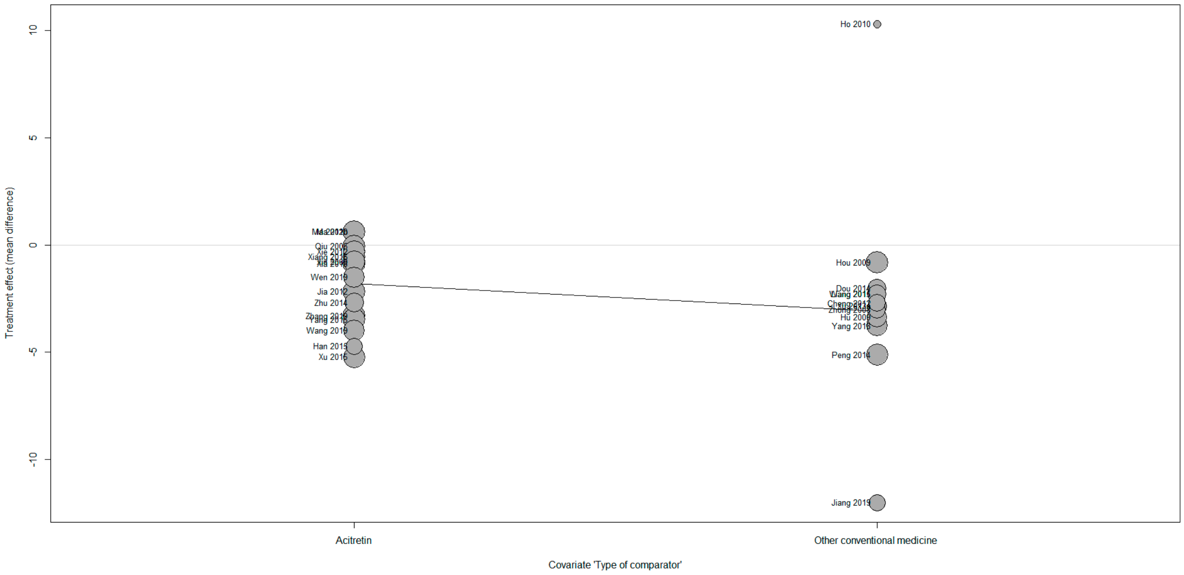

Supplementary Figure S3. Bubble plot of continuous PASI score for type of comparator.

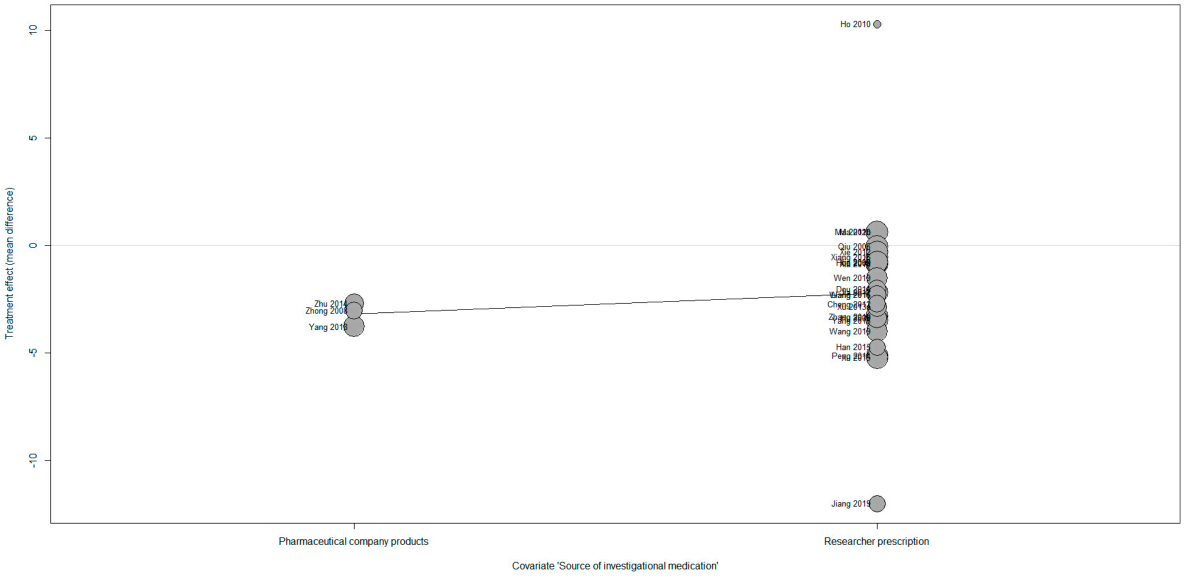

Supplementary Figure S4. Bubble plot of continuous PASI score for source of investigational medicine.

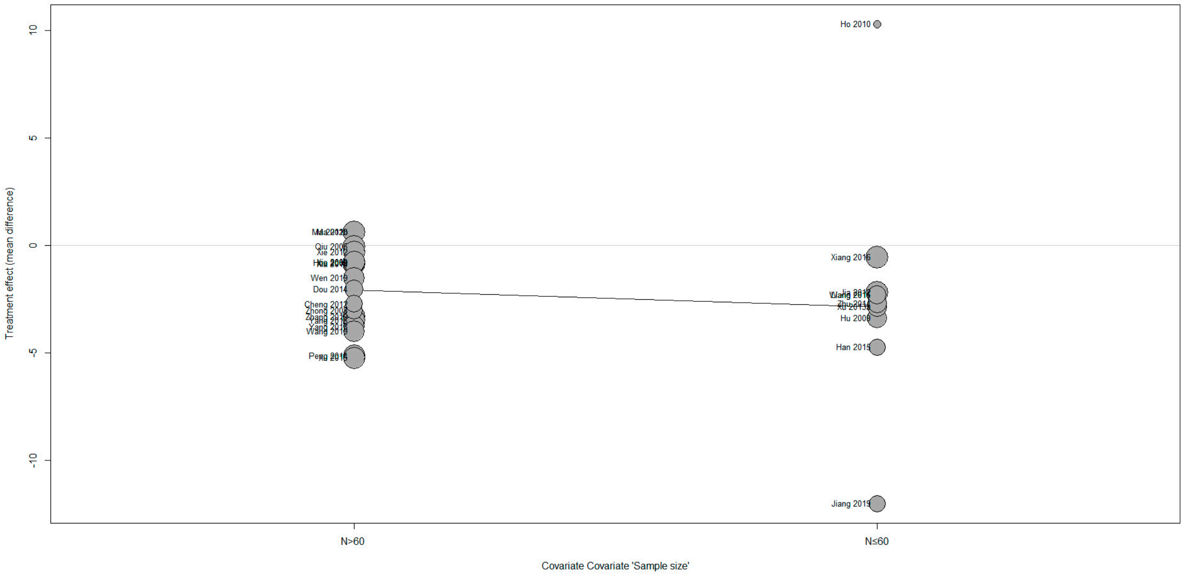

Supplementary Figure S5. Bubble plot of continuous PASI score for sample size.

Supplementary Table S2. Search terms used in each database

Medline

	Searches	Results
#1	Psoriasis[Mesh]	42404
#2	(Psoriases[Title/Abstract]) OR (Pustulosis of Palms[Title/Abstract] AND Soles[Title/Abstract]) OR (Pustulosis Palmaris et Plantaris[Title/Abstract]) OR (Palmoplantaris Pustulosis[Title/Abstract]) OR (Pustular Psoriasis of Palms[Title/Abstract] AND Soles[Title/Abstract])	188
#3	“Plants, Medicinal”[MeSH] OR “Drugs, Chinese Herbal”[MeSH] OR “Medicine, Chinese Traditional”[MeSH] OR “Medicine, Kampo”[MeSH] OR “Medicine, Korean Traditional”[MeSH] OR “Herbal Medicine”[MeSH] OR “Prescription Drugs”[MeSH] OR “traditional Korean medicine”[Title/abstract] OR “traditional Chinese medicine”[Title/abstract] OR “traditional oriental medicine”[Title/abstract] OR “Kampo medicine”[Title/abstract] OR herb*[Title/abstract] OR decoction*[Title/abstract] OR botanic*[Title/abstract]	234484
#4	#1 AND #2 AND #3	**283**

1

EMBASE

	Searches	Results
#1	'Psoriasis'/exp	106,080
#2	'Psoriases' OR 'Pustulosis of Palms and Soles' OR 'Pustulosis Palmaris et Plantaris' OR 'Palmoplantaris' OR 'Pustulosis' OR 'Pustular Psoriasis of Palms and Soles'	5857
#3	‘medicinal plant’/exp OR ‘medicinal plant’ OR ‘herbaceous agent’/exp OR ‘herbaceous agent’ OR ‘chinese medicine’/exp OR ‘chinese medicine’ OR ‘kampo medicine’/exp OR ‘kampo medicine’ OR ‘kampo medicine (drug)’/exp OR ‘kampo medicine (drug)’ OR ‘korean medicine’/exp OR ‘korean medicine’ OR ‘herbal medicine’/exp OR ‘herbal medicine’ OR ‘prescription drug’/exp OR ‘prescription drug’ OR ‘oriental medicine’/exp OR ‘oriental medicine’ OR ‘alternative medicine’/exp OR ‘alternative medicine’ OR ‘complementary medicine’ OR ‘herb’/exp OR ‘herb’ OR ‘decoction’ OR ‘botanic’	585795

#4	#1 AND #2 AND #3	**63**

CENTRAL

	Searches	Results
#1	MeSH descriptor: [Psoriasis] explode all trees	3442
#2	(“Psoriases” OR “Pustulosis of Palms and Soles” OR “Palmoplantaris Pustulosis” OR “Pustulosis Palmaris et Plantaris” OR “Pustular Psoriasis of Palms and Soles”):ti,ab,kw	11
#3	MeSH descriptor: [Plants, Medicinal] explode all trees	946
#4	MeSH descriptor: [Drugs, Chinese Herbal] explode all trees	3645
#5	MeSH descriptor: [Medicine, Chinese Traditional] explode all trees	1219
#6	MeSH descriptor: [Medicine, Kampo] explode all trees	46
#7	MeSH descriptor: [Medicine, Korean Traditional] explode all trees	33
#8	MeSH descriptor: [Herbal Medicine] explode all trees	63
#9	MeSH descriptor: [Prescription Drugs] explode all trees	108
#10	(“traditional Korean medicine” OR “traditional Chinese medicine” OR “Traditional oriental medicine” OR “Kampo medicine” OR herb* OR decoction* OR botanic*):ti,ab,kw	18819
#11	(#1 OR #2) AND (#3 OR #4 OR #5 OR #6 OR #7 OR #8 OR #9 OR #10) in Trials	**48**

OASIS

	Searches	Results
#1	건선 AND 한약	**5**

KISS

	Searches	Results
#1	건선 AND 한약	**3**

RISS

	Searches	Results
#1	건선 AND 한약	**2**

KCI

	Searches	Results
#1	건선 AND 한약	**7**

CNKI

	Searches	Results
#1	(TI='银屑病' OR '牛皮癣' OR '白疕' OR '寻常型银屑病' OR '点滴型银屑病' OR +'银屑病关节炎' OR '脓疱型银屑病' OR '关节病型银屑病') AND (TI='中药' OR '中医药' OR '中草药' OR '本草' OR '汤' OR '丸' OR '散' OR '方' OR '颗粒 OR '胶囊'	**1161**

	OR '自拟') AND (AB='银屑病' OR '牛皮癣' OR '白疕' OR '寻常型银屑病' OR '点滴型银屑病' OR '银屑病关节炎' OR '脓疱型银屑病' OR '关节病型银屑病') AND (AB='中药' OR '中医药' OR '中草药' OR '本草' OR '汤' OR '丸' OR '散' OR '方' OR '颗粒 OR '胶囊' OR '自拟') AND (AB='随机')	

Wanfang data

	Searches	Results
#1	题名:(“银屑病”or“牛皮癣”or“白疕”or“寻常型银屑病”or“点滴型银屑病”or “银屑病关节炎”or ”脓疱型银屑病”or ”关节病型银屑病”) and 题名:("汤” or “丸” or “散" or "中药") and 摘要:(“银屑病”or“牛皮癣”or“白疕”or“寻常型银屑病”or“点滴型银屑病”or “银屑病关节炎”or ”脓疱型银屑病”or ”关节病型银屑病”) and 摘要:("汤” or “丸” or “散" or "中药") and 摘要:("随机")	**813**

CiNii

	Searches	Results
#1	(“銀屑″ OR “Psoriasis” OR “Pustulosis of Palms and Soles” OR “Palmoplantaris Pustulosis” OR “Pustulosis Palmaris et Plantaris” OR “Pustular Psoriasis of Palms and Soles”) AND (“traditional Korean medicine” OR “traditional Chinese medicine” OR “Traditional oriental medicine” OR “Kampo medicine” OR herb OR decoction OR botanic OR 漢方薬 OR ハーブ OR 散 OR 汤 OR 丸)	**49**
